# Workload and workflow implications associated with the use of electronic clinical decision support tools used by health professionals in general practice: a scoping review

**DOI:** 10.1186/s12875-023-01973-2

**Published:** 2023-01-20

**Authors:** Emily Fletcher, Alex Burns, Bianca Wiering, Deepthi Lavu, Elizabeth Shephard, Willie Hamilton, John L. Campbell, Gary Abel

**Affiliations:** College of Medicine and Health, University of Exeter Medical School, St Luke’s Campus, Heavitree Road, Exeter, Devon EX1 2LU England

**Keywords:** General practice, Workload, Electronic clinical decision support, Consultations, Diagnosis, Risk

## Abstract

**Background:**

Electronic clinical decision support tools (eCDS) are increasingly available to assist General Practitioners (GP) with the diagnosis and management of a range of health conditions. It is unclear whether the use of eCDS tools has an impact on GP workload. This scoping review aimed to identify the available evidence on the use of eCDS tools by health professionals in general practice in relation to their impact on workload and workflow.

**Methods:**

A scoping review was carried out using the Arksey and O’Malley methodological framework. The search strategy was developed iteratively, with three main aspects: general practice/primary care contexts, risk assessment/decision support tools, and workload-related factors. Three databases were searched in 2019, and updated in 2021, covering articles published since 2009: Medline (Ovid), HMIC (Ovid) and Web of Science (TR). Double screening was completed by two reviewers, and data extracted from included articles were analysed.

**Results:**

The search resulted in 5,594 references, leading to 95 full articles, referring to 87 studies, after screening. Of these, 36 studies were based in the USA, 21 in the UK and 11 in Australia. A further 18 originated from Canada or Europe, with the remaining studies conducted in New Zealand, South Africa and Malaysia. Studies examined the use of eCDS tools and reported some findings related to their impact on workload, including on consultation duration. Most studies were qualitative and exploratory in nature, reporting health professionals’ subjective perceptions of consultation duration as opposed to objectively-measured time spent using tools or consultation durations. Other workload-related findings included impacts on cognitive workload, “workflow” and dialogue with patients, and clinicians’ experience of “alert fatigue”.

**Conclusions:**

The published literature on the impact of eCDS tools in general practice showed that limited efforts have focused on investigating the impact of such tools on workload and workflow. To gain an understanding of this area, further research, including quantitative measurement of consultation durations, would be useful to inform the future design and implementation of eCDS tools.

## Introduction

UK General Practitioners (GPs) manage a high and rising workload of increasingly complex patient care with many competing demands to attend to within time-limited consultations [1]. This, and ongoing recruitment and retention challenges, has led to a GP workforce ‘crisis’ [2–5]. The COVID-19 pandemic has introduced further pressures on general practice, with associated back-logs of consultations, diagnoses, and referrals [6–9]; GP workload therefore continues to be an increasingly pressing issue for health professionals, patients and policy makers.

Clinical decision support (CDS) tools are used by health professionals to assist with clinical decision making in relation to screening, diagnosis and management of a range of health conditions [10–14]. Many CDS tools exist for use in primary care and more recently are being embedded in electronic form (eCDS) within practice IT systems, drawing directly on data within patients’ electronic medical records (EMR) for their operation [11, 15, 16]. Many Clinical Commissioning Groups and Primary Care Networks have supported the introduction of eCDS tools that facilitate diagnosis and expedite referral for certain conditions, such as cancer, particularly since the COVID-19 pandemic [17]. For the purpose of this article, an eCDS tool is defined as any electronic or computerised tool which provides an output pertaining to a possible diagnosis and/or management of a health condition, using patient-specific information.

The workload implications of GPs using eCDS tools during consultations are unclear. One way of examining GP workload is to evaluate the duration of consultations [18], although that is only a single element of GP work, not including time taken for managing referrals, investigations, results, and general administration, undertaking training, and supervising colleagues [19, 20]. The duration of consultations and the ‘flow’ of patients through consulting sessions, however, provide key ways of measuring workload as these have an impact upon GPs’ levels of stress throughout the working day [21–23]. Understanding whether using eCDS tools impacts consultation duration and patient ‘flow’ through consulting sessions may help facilitate the implementation of eCDS tools into practice.

Here we aimed to establish if there is pre-existing evidence on potential workload, including impact on consultation durations, associated with the use of eCDS tools by health professionals in general practice and primary care. The objective of this literature review therefore was to identify the available evidence on using eCDS tools and analyse their impact on workload.

## Methods

A systematic scoping review was undertaken to identify literature using the stages set out in the Arksey and O’Malley methodological framework, enhanced by more recent recommendations [24, 25]. This method enables examination of the extent, range and nature of research activity with an aim of identifying all existing relevant literature.

A broad research question was used: What is known from the existing literature about the use of eCDS tools by health professionals in general practice/primary care and the associated impact on workload and patient ‘flow’ through consulting sessions?

**Fig. 1:**
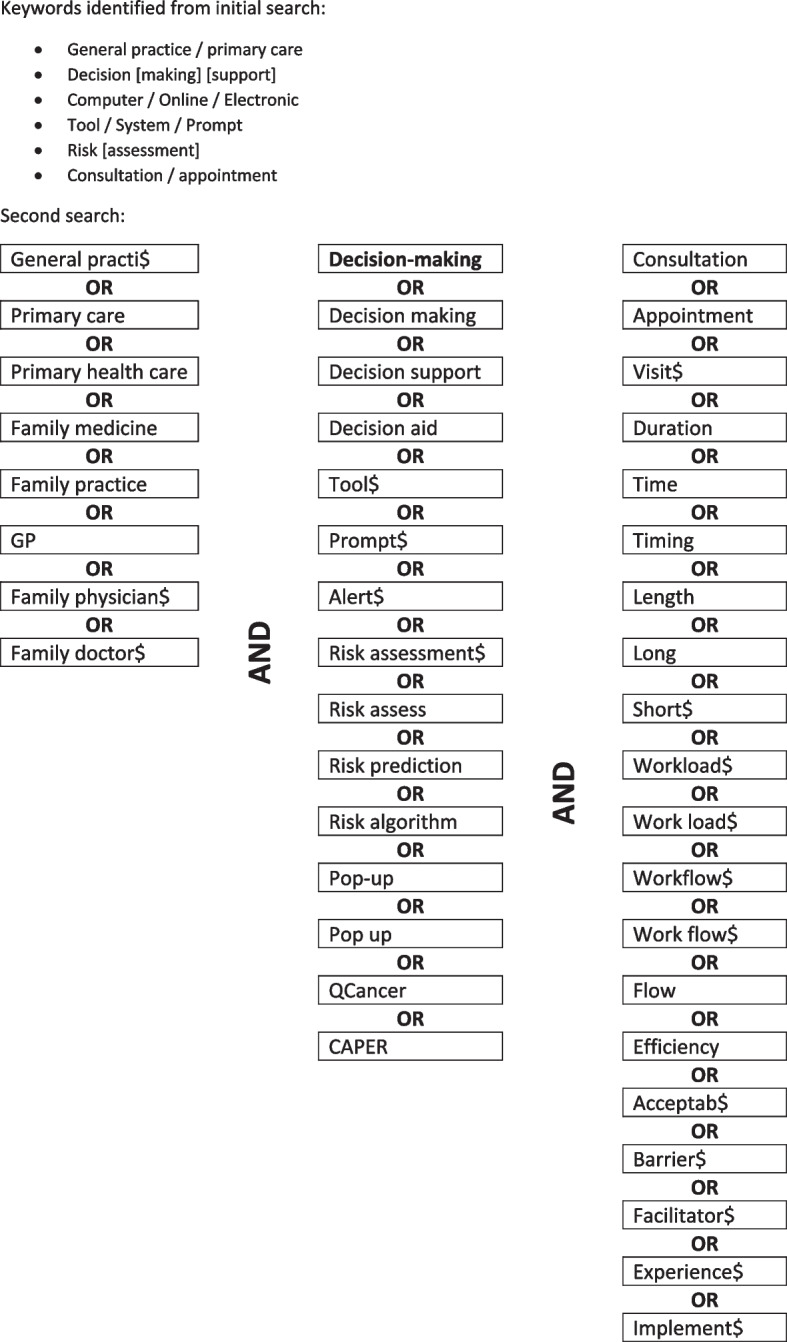
Search terms

An initial scoping search was conducted using the databases: MEDLINE (Ovid), HMIC (Ovid) and Web of Science (TR). Keywords from titles and abstracts identified by this search, and index terms used to describe these articles, were identified (see Fig. 1). A second search across the same databases was then undertaken using the identified keywords and index terms, and studies collated for abstract and title screening to identify relevant full-text articles to be reviewed. The searches were conducted in September 2019 and updated in August 2021. The review extensively targeted articles in written English, and published in a ten-year period prior to the initial search date. This time period was selected in order to identify research on eCDS in the context of today’s general practice and primary care, and to manage review in context of available resources. A comprehensive search strategy and set of search terms used is provided in Fig. 1.

The review aimed to identify research studies, reports and articles, including literature reviews, investigating the use of eCDS tools by all health professionals in relation to their impact on workload, such as consultation duration. The focus on ‘health professionals’ in primary care, not just on GPs, was intentional – we sought to identify all relevant contextual research. Therefore, studies concerning any type of health condition, eCDS tool, healthcare context within primary care or health professional were eligible. Both quantitative and qualitative evidence were included. Systematic reviews were included as studies in their own right, and thereafter the references of studies included in those reviews were screened for eligibility and relevance. Eligible and relevant references within a systematic review were then included in addition to those primary studies identified by the original searches. Studies relating specifically to the design or development of eCDS tools, and those focussing on clinical factors associated with specific conditions, were excluded. Protocol articles were excluded if the published results article of the same study were available.

Study selection was guided by: (i) an initial team meeting to discuss inclusion and exclusion criteria, (ii) all abstracts and full text articles were independently reviewed by two reviewers, and (iii) team meetings were held throughout the process to discuss and resolve conflicts of agreement. The following key information was gathered from the included studies: author(s), year of publication, study origin, study aims, type of eCDS tool in study, study population/context, methods, and outcome measures. EF, a health services researcher, classified the key findings into categories, defined as consultation duration-related (‘perceived’ or ‘objectively-measured’), or ‘other’ workload-related. The articles were organised using Covidence review software, then collated in a descriptive format using Microsoft Excel, and reviewed to summarise the key findings.

## Results

**Fig. 2:**
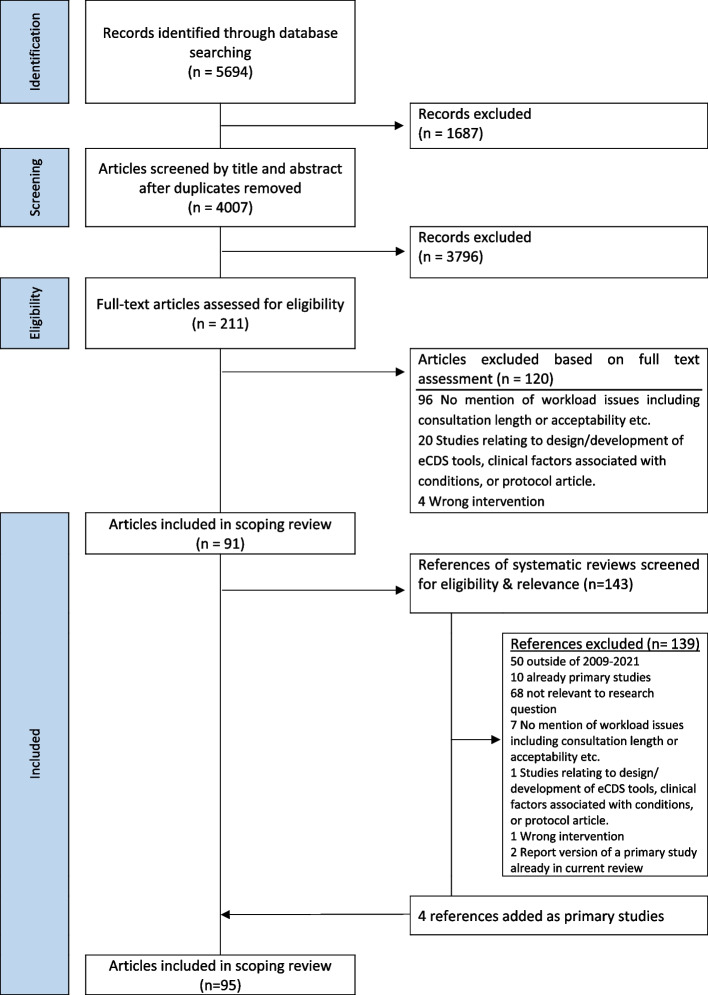
Summary of the screening process

The database search yielded 5694 publications (4007 after removal of duplicates, Fig. 2). After screening titles and abstracts, 211 publications were selected for full-text screening. Of these, 120 were excluded for not meeting the inclusion criteria, resulting in 91 publications being included in the scoping review. Four of these articles were systematic reviews; screening of eligibility and relevance of references included in those reviews led to the inclusion of a further four articles. The total 95 included articles referred to 87 research studies.

### Description of included articles

All studies were conducted in high-income countries, with the exception of one from Sub-Saharan Africa. A third of the articles from the studies originated in the USA (36), with UK and Australian articles comprising another third (21 and 11 respectively). A further 18 publications originated in Canada and mainland Europe, with the remaining studies conducted in New Zealand (2), South Africa (2) and Malaysia (1). For most articles workload was not the main focus, with only 16 examining it either as a main focus or as one of the aims.

The most common clinical areas of focus among the eCDS tools studied were cancer risk assessment (15 articles), cardiovascular disease (11), and prescribing for various conditions (10). Other common clinical areas included: blood-borne viruses (3 articles), and various other long-term conditions (14 articles, including those on diabetes, chronic kidney disease, asthma, Chronic Obstructive Pulmonary Disease, and hypertension). Smaller numbers focussed on tools for other conditions including: transient ischaemic attack and stroke, abdominal aortic aneurysm, respiratory infections, psychiatric disorders, skin conditions, hearing loss, and familial conditions (one or two publications on each). Some tools were also designed to support general delivery of care across a range of domains such as maternal and child health, occupational health, behavioural health, and geriatric home care.

A third of articles (31) utilised purely qualitative methods, almost all of which included interviews and/or focus groups with health professionals. One exception reported conversation analysis of audio- and video-recorded consultations and another study reported observations of consultations. Twenty-eight articles reported quantitative methods; 23 involved a survey of health professionals and/or analysis of EMR data or usage data from the investigated tool. The other quantitative articles included three randomised controlled trials and two observational studies. The remaining 28 articles utilised mixed methodologies. The majority of these involved either a survey of health professionals plus qualitative interviews/focus groups (*n* = 12) or an analysis of EMR/tool usage data in addition to qualitative interviews, focus groups and/or observations (*n* = 15). Four further articles were systematic reviews, two involving qualitative synthesis and one being a mixed-methods narrative review. All included articles are summarised in the data extraction table (Table 1).

**Table 1:** Data extraction table

Authors	Origin	Aims	Context	Methods	Outcome measures	Key findings of interest	Impact on time/workload
Ahmad et al. 2010 [26]	Canada	To enhance understanding about computer-assisted health-risk assessments (HRA) from physicians’ perspectives regarding benefits and concerns/challenges	**Condition of focus**: Domestic violence **Setting**: 1 family practice clinic **Tool**: Health Risk Assessment tool - Embedded/linked with EMR: Yes - Interruptive alert: No, guides visit - User-driven: Physician - Risk score: No	Qualitative interviews	(1) What do you think of your experience with the HRA? (2) How would you describe its potential across various risks and visits? (3) Would you recommend such HRA in a family practice setting? 4) What factors are important for its implementation in a family practice setting?	Theme 2: perceived risks & challenges (subtheme: length of visit) - Some expressed concern about the increase in length of the visit - Others managed the time pressure by offering follow-up visits or viewed the task of risk review as a professional obligation even if it meant increases in the consultation time - Follow-up visit offered in order to avoid “taking time away” from other waiting patients - ‘Patient readiness'—not always the right time to address in the visit	Perception **Impact on time**: Mixed views
Arts et al. 2017 [27]	Netherlands	To investigate the effectiveness of a CDSS as measured by GPs' adherence to the Dutch GP guideline for patients with Atrial Fibrilation	**Condition of focus:** Stroke prevention in AF **Setting**: General Practice **Tool**: CDS using antithrombotic guidelines - Embedded/linked with EMR: Yes - Interruptive alert: Yes - User-driven: Physician - Risk score: Yes	Quantitative RCT	**Primary**: Difference in the proportion of patients with AF treated in accordance with the guideline between the intervention and control groups **Secondary**: reasons GPs provided for deviating from the guideline and how they responded to required justification	Limited evidence for effectiveness, attributed to low usage Reasons for low usage discussed in a separate qualitative evaluation, but included barriers relating to lack of time, too many alert notifications and tool functionality limitations	Perception **Impact on time:** Increase **Driving perception**: lack of time, too many alerts
Arts et al. 2018 [28]	Netherlands	To identify remediable barriers which led to low usage rates seen in RCT	**Condition of focus**: Stroke prevention in AF **Setting**: General Practice **Tool**: CDS using antithrombotic guidelines - Embedded/linked with EMR: Yes - Interruptive alert: Yes - User-driven: Physician - Risk score: Yes	Mixed: quantitative survey + focus group	Barriers and facilitators for CDSS use	More than 75% of GPs answered the question: "What were the most important reasons for not opening the recommendations?" citing reasons relating to lack of time Many felt there was lack of time during the appointment to perform the suggested actions. Some GPs scheduled follow up appointments for this purpose	Perception **Impact on time**: Increase
Baron et al. 2017 [29]	USA	To evaluate the value and feasibility of three examples of CDS relating to occupational health in five primary care group practices	**Condition of focus:** Occupational Health **Setting**: Primary Care **Tool**: CDS using guidelines - Embedded/linked with EMR: Yes - Interruptive alert: No - User-driven: Physician - Risk score: No	Qualitative:- interviews + observations	**Interviews**: - physicians' daily work patterns - experience with EMRs and CDS - attitudes and practice regarding consideration of health factors encountered in a patient’s job - how patients' work data is collected in the EMR **Observations**: - workflow data through observations of clinic staff	The amount of clinical time the CDS tools would require was a prominent concern 1 of 7 themes: clinician acceptance is affected by whether CDS adds or saves time	Perception **Impact on time:** No conclusion
Bauer et al. 2014 [30]	USA	To examine the attitudes and opinions of paediatric users’ toward the Child Health Improvement through Computer Automation (CHICA) system	**Condition of focus**: Child Health **Setting**: Community paediatric clinics **Tool**: Child Health Improvement (CHICA) CDS for paediatric visits - Embedded/linked with EMR: Yes - Interruptive alert: No, guides visit - User-driven: Physician & patient - Risk score: No	Quantitative: survey + free text	General acceptability and satisfaction	Critical opinions of CHICA were that it wasted time and money. This perception persisted in spite of informal time-flow studies in one of the clinics showing that CHICA did not create significant delays Approximately half of respondents reported that it did slow down clinic	Perception and objective measure showed conflict **Impact on time:** Increase (perception), no impact (objective) **Driving perception:** lack of time, workflow disruption
Carlfjord et al. 2011 [31]	Sweden	To explore how staff at 6 Primary Health Care units experienced implementation of a tool for lifestyle intervention in primary health care	**Condition of focus:** Preventive care **Setting**: PHC units **Tool**: CDS for lifestyle intervention & preventive services - Embedded/linked with EMR: No - Interruptive alert: No - User-driven: Patient-completed - Risk score: Unclear	Qualitative: focus groups + interviews	- Overall working situation coinciding with the implementation process - Experiences of implementation activities and the tool - How to address lifestyle issues with patients - Openness to innovations	GPs' perception of workload already being too heavy and accommodating never-ending changes such as the lifestyle intervention tool may threaten independence and bring extra work	Perception **Impact on time:** Increase **Driving perception:** workload already heavy
Carlfjord et al. 2012 [32]	Sweden	Qualitative evaluation of the 2011 study to explore staff’s perceptions of handling lifestyle issues, including the consultation as well as the perceived usefulness of the tool	**Condition of focus:** Preventive care **Setting**: PHC units **Tool**: CDS for lifestyle intervention & preventive services - Embedded/linked with EMR: No - Interruptive alert: No - User-driven: Patient-completed - Risk score: Unclear	Qualitative: focus groups	Staff responses to two scenarios: - How to handle the patient/consultation - Advice to give to another clinic considering implementing the tool	Lifestyle issues tend to be forgotten when the workload is increasing, despite interest and awareness of its importance Many staff members found it difficult to initiate a conversation about lifestyle, particularly alcohol consumption, when the patient is seeking care for something else Time is too pressured to be focused on lifestyle/prevention especially in acute times/low resources	Perception **Impact on time:** Increase **Driving perception:** no time for preventive care
Chiang et al. 2017 [33]	Australia	To test the acceptability and feasibility of the Treat to Target CVD (T3CVD), an EMR-based CDS tool, for the evaluation of Absolute CVD Risk in general practice	**Condition of focus:** Cardiovascular Disease **Setting**: 1 general practice **Tool**: CDS for CVD risk assessment - Embedded/linked with EMR: Yes - Interruptive alert: Yes - User-driven: Automatic, based on EMR data - Risk score: Yes, for patients with > 10% risk of CVD	Qualitative interviews	Acceptability and feasibility of the T3CVD tool, including GPs’ experiences with the tool in real-world clinical practice, under a framework of 3 themes: - patient control - clinical quality of care - technology capability/capacity	GPs described how the ACVDR assessment pop-up necessitated additional time, often needing to arrange a follow-up visit if there was no time to discuss While the tool had capacity to save time by automating information provision rather than GPs manually accessing the existing CVD risk tool, it is potentially disruptive and adds to many existing pop-ups	Perception **Impact on time**: Mixed views
Chiang et al. 2015 [15]	Australia	To explore the use of a cancer risk tool, which implements the QCancer model, in consultations and its potential impact on clinical decision making	**Condition of focus:** Cancer **Setting:** General Practice **Tool:** QCancer risk tool - Embedded/linked with EMR: No - Interruptive alert: No - User-driven: GP - Risk score: Yes, for each of 10 cancers	Qualitative: simulated consultations + interviews	1. Coherence 2. Cognitive participation 3. Collective action 4. Reflexive monitoring	Tool was easy and quick to use, but introducing the emotive topic of cancer caused anxiety. Risk output 'too confronting' to use in a consultation and could lead to a loss of control over the consultation and time being used to reassure which may lead to overrunning by 20-30 m GPs thought pop-up alerts would add to alert fatigue	Perception **Impact on time:** Increase **Driving perception:** impact on conversation
Crawford et al. 2011 [34]	Scotland	To understand primary care practitioners’ views towards screening for diabetic foot disease and their experience of the SCI-DC system	**Condition of focus:** Diabetic foot disease **Setting:** General Practice **Tool:** CDS for screening - Embedded/linked with EMR: No - Interruptive alert: No - User-driven: GP - Risk score: Unclear	Qualitative interviews	Views on and use of decision support systems, specifically SCI-DC	The duplication of effort to complete the SCI-DC and then the GP IT system through which the practice receives remuneration is unnecessarily time consuming. Integration into GP IT systems is central to its adoption	Perception **Impact on time**: Increase **Driving perception:** stand-along system, double data entry needed
Curry et al. 2011 [35]	Canada	To explore two issues in the implementation of CDS for Diagnostic Imaging: - Will physicians incorporate decision-support technology into their clinical routines? - Will physicians follow clinical advice when Provided?	**Condition of focus:** Diagnostic imaging **Setting:** Family Medicine clinic **Tool:** CDS to guide decisions to order imaging **-** Embedded/linked with EMR: Yes - Interruptive alert: No - User-driven: GP - Risk score: No	Mixed: quantitative analysis of useage data + qualitative interviews	**Quantitative**—usage by clinicians **Qualitative**—perceived effects of taking part in the study and challenges	The largest challenge was perceived interference with usual work flows, specifically the interactivity between EMR and the CDS (perceived to be too slow, although measured as less than 1 s). The time for physicians to interact with CDS was also perceived to be too long Time taken to use tool (described in Methods) 2 min 15 s as follows: - 1 min to enter data, 5 s for CDS to check if appropriate - < 1 min for clinician to look at recommendation if not appropriate - 10 s to complete DI order if still required	Perception and objective measure of time to use tool showed conflict **Impact on time**: Increase **Driving perception:** slow software, workflow disruption
Dikomitis et al. 2015 [36]	UK	To collect data on the (non)use of electronic risk assessment tools (eRATs) and on the experiences of using the tool in everyday practice	**Condition of focus:** Cancer **Setting:** General Practice **Tool:** eRATs - Embedded/linked with EMR: Yes - Interruptive alert: Yes - User-driven: GP - Risk score: Yes	Qualitative interviews	Normalisation Process Model: (1) interactional workability (2) relational integration (3) skill-set workability (4) contextual integration	Interactional workability—GPs' reactions to the on-screen prompts was influenced by different factors: - the approach of the doctor - the GP’s clinical experience - time pressures in specific consultations Contextual integration—most issues related generally to new interventions being implemented: - GPs expected to multi-task within one consultation - constant time pressures - prompt fatigue is a risk if added to an already 'busy screen' and alerts may not be responded to - concern over workload for secondary care	Perception **Impact on time**: Increase **Driving perception:** lack of time
Duyver et al. 2010 [37]	Belgium	To explore GPs' perceptions of feasibility and added value of the MDS-HC as a geriatric assessment tool and to investigate potential barriers and facilitating factors regarding the implementation of this tool in an ambulatory community setting	**Condition of focus:** Geriatric care **Setting:** General Practice **Tool:** Home care assessment tool - Embedded/linked with EMR: No - Interruptive alert: No - User-driven: GP - Risk score: No	Quantitative: survey + free text	Four assessment areas: (1) technical acceptability (2) clinical relevance of the tool (3) management and optimization of health care planning (4) valorisation of the role of the GP	Free comments from GPs: - Long and fastidious encoding - CDS was too long, added admin workload - Excellent concept, worth making easier and shorter to use	Perception **Impact on time**: Increase **Driving perception:** slow software
Eaton et al. 2012 [38]	USA	To examine Abdominal Aortic Anneurysm (AAA) screening ordering in an academic primary care internal medicine clinic that uses physician reminders based on real-time CDS for preventive screening and to identify why screening ordering rates vary among providers	**Condition of focus:** Abdominal Aortic Aneurysm (AAA) **Setting:** Primary Care clinic **Tool:** CDS for AAA screening - Embedded/linked with EMR: Yes - Interruptive alert: Yes - User-driven: GP - Risk score: No	Quantitative: records review	Features of the first visit during the study: - visit date - visit type (general medical examination vs. other type) - provider role (staff physician vs. other) - provider gender - was AAA screening ultrasound was ordered during the visit	Visit time (based on the fixed length of different visit types) is an important determinant for preventive screening Patients more likely to have screening ultrasound ordered during longer medical examinations, which usually has more time allotted (40 min) and often has a disease-prevention component During longer medical examinations, 24% of eligible patients had the recommended AAA screening ordered, compared with only 6% during shorter visits	Objective measure of time for whole consultations (via proxy of visit type) **Impact on time:** Increase
Laforest et al. 2019 [39]	UK	To review the tools available, clinician attitudes and experiences, and the effects on patients of genetic cancer risk assessment in general practice	**Condition of focus:** Cancer **Setting:** General Practice **Tool:** range of risk assessment tools, including web-based, risk-stratification, and paper-based - Embedded/linked with EMR: Range - Interruptive alert: Range - User-driven: GP - Risk score: Range	Systematic review	1. What tests/tools are available to identify increased genetic risk of cancer in general practice? 2. What are clinicians’ attitudes towards Screening/testing population groups for genetic cancer risk? 3. What are the levels of patient knowledge, satisfaction, and anxiety in relation to tests and communication by a GP about cancer risk? 4. What are patients’ risk perceptions following screening/testing for genetic cancer risk in primary care? 5. What are the outcomes of referrals following genetic cancer risk identification in general practice?	5 studies examined GP attitudes: - Owens et al. (43): some providers concerned over the time needed to counsel patients who were newly determined as at high risk, and regarding liability for not successfully providing risk counselling - Wu et al. (45): physicians at two primary care clinics felt they were already collecting high-quality family histories and that the tool would negatively impact their workflow. Physicians believed that patients would redirect discussions away from physician priorities and, instead, focus on tool recommendations. However, post-implementation, 86% of physicians found the tool improved their practice, and none reported adverse effects on workflow GPs were worried about the impact of risk assessment on patient anxiety, particularly if discussions with whole families would be required. GPs were concerned about their ability to explain risk and implications in short, routine appointments	Perception **Impact on time:** Mixed views
Fathima et al. 2014 [40]	Australia	To systematically review randomized controlled trials evaluating effectiveness of CDS in the care of people with asthma and COPD and to identify the key features of those systems that have the potential to overcome health system barriers and improve outcomes	**Condition of focus:** Asthma & COPD **Setting:** Primary care **Tool:** range of CDS systems, including prevention & management, providing guidelines - Embedded/linked with EMR: Range - Interruptive alert: Range - User-driven: Clinician - Risk score: No	Systematic Review (qualitative syntheses)	Assessment of intervention effects 1. Type of CDS intervention 2. Effectiveness of CDS: - Clinical outcomes - Healthcare process measures - User workload and efficiency outcomes - Relationship-centred outcomes - Economic outcomes - Use and Implementation outcomes	Workload and efficiency outcomes assessed included asthma documentation by ED doctors, consultation time, time for disposition decision in the ED, and user knowledge These were assessed as the primary outcome by two trials, of which one trial showed significant improvement in rate of asthma documentation. The other trial did not show any effect from the use of CDS on the time taken to make a disposition decision 83% of studies of CDS tools which were stand-alone in design had favourable clinical outcomes, compared with 38% of embedded designs. This may be due to alert fatigue and too low a threshold for alerts being generated Low evidence provided by studies re user workload & efficiency. One RCT in a hospital ED measured consultation time as a marker of workflow efficiency, finding no significant difference in consultation time between the intervention group compared with control	Objective measure of time for whole consultations (1 study in a systematic review) **Impact on time:** neither increase nor decrease
Finkelstein et al. 2017 [41]	USA	To implement a comprehensive informatics framework to promote breast cancer risk assessment and chemoprevention in primary care that was informed by potential user feedback (usability testing to determine barriers and facilitators affecting the toolbox use by providers)	**Condition of focus:** Cancer **Setting:** Primary care **Tool:** Breast cancer risk assessment & chemoprevention - Embedded/linked with EMR: Yes - Interruptive alert: No - User-driven: Clinician & patient - Risk score: Yes	Qualitative interviews	Ease of use, content, navigation	Ease of use: notifications were noted to be too time consuming to process	Perception **Impact on time:** Increase **Driving perception:** Lack of time
Fox et al. 2014 [42]	USA	To evaluate adherence to an evidence-based Chronic Kidney Disease computer decision-support checklist in patients treated by Primary Care Physicians compared with usual care at a single site	**Condition of focus:** Chronic Kidney Disease **Setting:** Primary care clinic **Tool:** CDS checklists for CKD - Embedded/linked with EMR: Unclear - Interruptive alert: No, guides visit - User-driven: Clinic staff - Risk score: No	Quantitative records review	Clinical measures of CKD management	Comment that the checklist was used to create a priority and incorporated into workflow so that CKD was treated appropriately. This is a step above a simple alert at the point of care and circumvented alert fatigue The 'extra time' needed for PCP to improve CKD care did not seem to adversely affect other areas of preventive care	Perception **Impact on time:** Neither increase nor decrease **Driving perception:** did not take time away from other areas of preventive care
Gill et al. 2019 [43]	USA	To examine the impact of Point Of Care CDS on diabetes management in small- to medium- sized independent primary care practices that had adopted the PCMH model of care	**Condition of focus:** Diabetes **Setting:** Primary care practices **Tool:** CDS for diabetes management - Embedded/linked with EMR: Yes - Interruptive alert: No, guides visit - User-driven: Automatic - Risk score: No	Mixed: quantitative analysis of EMR data + qualitative interviews	**Quantitative**: Clinical measures of diabetes management **Qualitative**: Barriers to and facilitators of successful implementation of CDS that achieved optimal diabetes management	Barriers impeding implementation of the CDS included time and reimbursement in light of the need for time to implement team-based care, not specifically regarding the impact of the CDS on visit lengths	Perception **Impact on time**: No conclusion
Green et al. 2015 [36, 44]	UK	To explore GPs’ experiences of incorporating Risk Assessment Tools (RATs) for lung and bowel cancers into their practice and to identify constraints and facilitators to the wider dissemination of the tools in primary care	**Condition of focus:** Cancer **Setting:** General Practice **Tool:** paper-based RATs - Embedded/linked with EMR: No - Interruptive alert: No - User-driven: Clinician - Risk score: Yes	Qualitative interviews	GPs' experiences of the implementation process and their use of the RATs in practice	A minority of participants did not feel RATs added to practice: "I’m not sure it fits into the consultation in a natural way of making a decision about the management of that patient. It’s one more thing to fit into a busy ten minute consultation”	Perception **Impact on time:** Increase **Driving perception:** workload already heavy
Gregory et al. 2017 [45, 46]	USA	To examine asynchronous alert-related workload in the EMR as a predictor of burnout in primary care providers (PCPs), in order to inform interventions targeted at reducing burnout associated with alert workload	**Condition of focus:** Generic **Setting**: Primary care **Tool:** inbox-style EMR alerts - Embedded/linked with EMR: Yes - Interruptive alert: No - User-driven: Clinician - Risk score: No	Mixed: quantitative survey + focus groups	Subjective alert workload (perception of time available to complete tasks) Objective alert workload (actual hours spent) Burnout (scale)	**Quantitative**: subjective alert workload was positively related to 2/3 dimensions of burnout. Subjective alert workload was also generally predictive of burnout, whereas objective alert workload was not This suggest that it is the perception of alert burden that predicts burnout, rather than the actual amount of time spent attending to alerts **Qualitative**: time spent managing alerts was a major theme in focus group discussion and survey comments	Perception and objective measure of workload showed conflict **Impact on workload**: Mixed views
Harry et al. 2019 [47]	USA	To identify adoption barriers and facilitators before implementation of CDS for cancer prevention in primary care	**Condition of focus:** Cancer **Setting:** Primary care clinics **Tool:** CDS for cancer prevention & screening - Embedded/linked with EMR: Yes - Interruptive alert: No, guides visit - User-driven: Clinician - Risk score: Unclear	Qualitative interviews	1. Factors that facilitate or hinder key informant support for the intervention 2. Key informant knowledge and beliefs about the intervention and tension for change 3. The relative advantage(s) of the intervention compared with other interventions currently available in the EMR 4. Relevant organizational culture norms and values related to cancer prevention and screening 5. Factors that may foster adoption from a key informant perspective 6. Related external policies and incentives 7. Implementation climate	PCP time limitations were a major concern. PCPs are being asked to do more with less time, including seeing more patients in a day, making some PCPs wonder how to fit the CDS into the visit It was perceived to be 5–10 min to use the tool, which would add time pressure as appointments are usually 'already 20 min behind' However, some same informants who mentioned time constraints also said that this would only be a limitation until PCPs learned the CDS tools	Perception **Impact on time:** Increase **Driving perception:** Lack of time
Hayward et al. 2013 [48]	UK	To understand how GPs interact with prescribing CDS in order to inform deliberation on how better to support prescribing decisions in primary care	**Condition of focus:** Prescribing **Setting**: General Practice **Tool:** CDS for prescribing - Embedded/linked with EMR: Yes - Interruptive alert: Yes - User-driven: GP - Risk score: No	Mixed: quantitative analysis of useage data + conversation analysis	Timing of computer tasks and utterances Prescribing alerts and responses Conversation analysis	Total mean duration of consultation: 9 min 3 s - time before prescribing = 5 min 47 s - during prescribing = 1 min 47 s - time after prescribing = 1 min 30 s Timing of alerts was problematic as they interrupt in order to correct decisions already made rather than to assist earlier deliberations. By the time an alert appears the GP will have potentially spent several minutes considering, explaining, negotiating, and reaching agreement with the patient, possibly given instructions, and printed information about treatment. An alert in the final seconds of the task increases the probability of it being ignored	Objective measure of time of whole consultations **Impact on time:** neither increase nor decrease
Henderson et al. 2013 [49, 50]	UK	To determine uptake of online diagnostic CDS and impact on clinical decision-making and patient management and to elicit users’ views of utility	**Condition of focus:** Generic **Setting**: General Practice **Tool**: online diagnostic CDS - Embedded/linked with EMR: No - Interruptive alert: No - User-driven: GP - Risk score: No	Mixed: focus group + survey	Whether and how well the system had been embedded in everyday practice, based on the evidence available from the focus groups and post-use survey	Low usage reported There was conflict at the organisational level, as agreement to participate in the study had been primarily by practice managers, while CDS use was to be by clinicians. This was linked to the major issue of no time having been identified for clinicians to use the system during a consultation Searches took 4 min within a 10 min consultation: ‘We have so many things thrown at us…the PCT telling us to do this and that you can get a little overwhelmed.’ (GP)	Perception and objective measure of time to use tool **Impact on time:** Increase **Driving perception:** workload already heavy
Heselmans et al. 2012 [51]	Belgium	To assess users’ perceptions towards the implemented EBMeDS, the investigation of user interactions with the system and possible relationships between perceptions and use	**Condition of focus:** Generic **Setting**: Family Practice **Tool**: CDS for a range of conditions - Embedded/linked with EMR: Yes - Interruptive alert: Yes - User-driven: GP - Risk score: No	Mixed: quantitative survey + qualitative interviews	**Qualitative**: factors that may account for acceptance and use of EBMeDS **Quantitative**: computer-recorded user interactions with the system over evaluation period of 3 months to assess the actual use of the system	Although majority of FPs were positive about the system, the most important reasons to neglect reminders related to the number of reminders and lack of time to read them (44%). However, 35% reported they could perform their tasks faster using the system Quantitative analysis of physicians’ log files:- Study measured number of seconds for a reminder to be closed after appearing, but this is only referred to in the discussion: - reminders open for less than 3 s were assumed to have been 'clicked away' (ie ignored) - 49% of alerts were open for < 2 s and 32.5% open for < 3 s which suggests that sensitivity threshold may have been too low	Perception **Impact on time:** Mixed views
Hirsch et al. 2012 [52]	Germany	To evaluate the uptake of an interactive, transactional, and evidence-based library of decision aids and its association to decision making in patients and physicians in the primary care context	**Condition of focus:** Range **Setting**: Primary Care **Tool**: CDS for a range of conditions, including CVD, AF, CHD, diabetes and depression - Embedded/linked with EMR: No - Interruptive alert: No - User-driven: GP - Risk score: No	Quantitative survey	Which module was used and how detailed the steps of the shared decision making process were discussed using a four point scale Physicians were asked who made the decision at the end of the consultation, and for a subjective appraisal of consultation length (“unacceptably extended”, “acceptably extended”, “neither nor”, “shortened”)	Subjective appraisal of consultation length: in 8.9% of consultations physicians said that they were “unacceptably extended” by the CDS, 76.3% of consultations were “acceptably extended”, 14.2% “neither nor”, and 0.5% were “shortened” Majority of physicians stated that the consultation length was either not extended or ‘acceptably’ extended Log files analysis reported average consulting time was 8 min, so use of CDS was therefore not extending the usual 10 min appointment slot	Perception **Impact on time**: Mixed views
Holt et al. 2018 [53]	UK	To identify the barriers to automated stroke risk assessment linked to invitations and screen reminders in primary care (AURAS-AF)	**Condition of focus:** Stroke prevention in Atrial Fibriliation **Setting**: General Practice **Tool**: stroke risk assessment - Embedded/linked with EMR: Yes - Interruptive alert: Yes - User-driven: GP - Risk score: Yes	Mixed: quantitative analysis of useage data + interviews	**Quantitative**: coded data indicating the responses to the screen prompts **Qualitative**: Researcher-led issues around AURAS-AF, and allowed people to express their own experiences and priorities	Time available and the patient's own agenda dictated whether the alert was used to introduce the topic into the consultation. In some cases, GPs recognised that the timing was not right to initiate a discussion GP estimated the alert added 5–10 min more on the consult ('so you leave it')	Perception **Impact on time:** Increase **Driving perception:** impact on conversation
Hoonakker et al. 2012 [54]	USA	To examine barriers, and possible improvements to a tool, HeartDecision (HD)	**Condition of focus:** Cardiovascular disease **Setting**: Primary care **Tool**: cardiac risk assessment - Embedded/linked with EMR: Yes - Interruptive alert: No - User-driven: GP - Risk score: Yes	Mixed: quantitative time study + survey + qualitative interviews + observations	A stop watch was used to measure the time the physicians spent on the different pages of the tool Survey: additional information about the need for such tools, use of the tool, barriers against its use, facilitators, and possible improvements	The time study showed that on average, physicians spent 13 min using the tool, which is 'too long' for a regular patient visit, which lasts on an average 10 min	Objective measure of time to use tool **Impact on time**: Increase
Kortteisto et al. 2012 [55]	Finland	To assess and describe in depth the specific reasons for HPs using or not using the eCDS in primary care	**Condition of focus:** Generic **Setting**: Primary care clinic **Tool**: CDS for a range of conditions - Embedded/linked with EMR: Yes - Interruptive alert: Yes - User-driven: GP - Risk score: No	Mixed: focus group + survey	**Focus groups**: general ideas about the eCDS, experiences of the use, practical problems, advantages /disadvantages for work, barriers to use and facilitators, and development issues **Survey**: system’s capacity and quality, as well as its perceived usefulness and ease of use	Common barrier was busy practice in primary care ‘When I am busy, I don’t look for anything really.’ ‘Nothing more than simply doing what I have to do.’ Within 'functionality', majority of clinicians reported it was 'rapid enough' Within ‘usefulness’, drug alerts 'motivate' but 'take time' and 'requires more time for paperwork'	Perception **Impact on time:** Mixed views
Krog et al. 2018 [56]	Denmark	To explore facilitators and barriers to using the eMDI in psychometric testing of patients with symptoms of depression in Danish general practice	**Condition of focus:** Depression **Setting**: General Practice **Tool**: CDS for diagnosis/monitoring of patients with depression - Embedded/linked with EMR: Yes - Interruptive alert: No - User-driven: GP - Risk score: Yes	Qualitative interviews	Determinants for using the eMDI in relation to the GPs’ capability, opportunity and motivation to change clinical behaviour	eMDI was a 'timesaver' compared to the paper version because of cutting out need for data entry or printing and scanning which frees up time for other tasks, e.g. more time for dialogue in the consultation (i.e. 'better' consultations through improved use of consultation time and prioritisation of GPs' time) However, for some interviewees, time and efficiency aspects have worked as a barrier because of lack of time to change routines and experiences with the eMDI as being too time-consuming when filled in during the consultation	Perception I**mpact on time:** Mixed view
Lafata et al. 2016 [57]	USA	To evaluate the association of exam room use of EMRs, HRA tools, and self-generated written patient reminder lists with patient–physician communication, recommended preventive health service delivery, and visit length	**Condition of focus:** Generic **Setting**: Primary care **Tool**: range of tools, including EMR tools, risk assessment tools and written patient lists - Embedded/linked with EMR: Range - Interruptive alert: Range - User-driven: Range - Risk score: n/a	Quantitative: observational	1. Visit length (face-to-face interaction time in minutes between patients and physicians); 2. Patient engagement communication behaviour; 3. Physician–patient-centred communication behaviour; and 4. Physician delivery of evidence-based preventive health services	On average, physicians spent almost 27 min with the patient (SD = 10 min) Mean visit length was longer for patients who used a self-generated written reminder list compared to patients who did not use such a list (30.0 vs. 26.5 min). Visit length was also significantly longer when the EMR was accessed in the exam room compared to those visits in which the EMR was not accessed in the exam room (27.7 vs. 23.9 min) Visits that included exam room–based use of the EMR lasted, on average, just over 3 min more than visits in which the EMR was not accessed in the exam room The use of a HRA instrument was not associated with increased visit length, but was not associated with decreased length either	Objective measure of time of whole consultations **Impact on time**: neither increase nor decrease
Litvin et al. 2012 [58]	USA	To describe use of the CDS, as well as facilitators and barriers to its adoption, during the first year of the 15-month intervention	**Condition of focus:** Prescribing **Setting**: Primary care practices **Tool**: CDS for antibiotic prescribing for Acute Respiratory Infections - Embedded/linked with EMR: Yes - Interruptive alert: No - User-driven: Physician - Risk score: No	Mixed: quantitative analysis of EMR data + qualitative interviews and observations	Using EMR data, CDS use was calculated at the practice level as the number of encounters at which an ARI diagnosis (or multiple diagnoses) using the CDS was made divided by the number of all encounters at which an ARI diagnosis was made, regardless of CDS use Qualitative data were recorded during practice site visits using a structured site visit field note Group interviews with staff elicited facilitators and barriers of CDS adoption	Organisational factors: - Barrier: 'use of CDS alters workflow' - Facilitator: perception that CDS speeds up the visit and shortens documentation time. Others felt that the CDS did not affect the length of the visit. None reported that the CDS slowed the visit	Perception **Impact on time**: Mixed view
Lugtenberg et al. 2015 [59]	Netherlands	To investigate the exposure to and experiences with the CDS quality improvement intervention, to gain insight into the factors contributing to the intervention’s impact	**Condition of focus:** Generic **Setting**: General Practice **Tool**: CDS for range of activities, including patient data registration, prescribing and management - Embedded/linked with EMR: Yes - Interruptive alert: Yes - User-driven: GP - Risk score: No	Mixed: quantitative analysis of usage data + survey + qualitative interviews	**Quantitative**: - NHGDoc data to measure exposure to the intervention in both study groups - Survey data on exposure to and experiences with the CDS intervention **Qualitative**: range of barriers that GPs and PNs perceive in using NHGDoc or similar CDS in practice	Survey: - Limited time available during and after consultation (60% of GPs and 16% of PNs) - Too much additional work required during and after consultation (60% of GPs, 27% of PNs)	Perception **Impact on time:** Increase **Driving perception:** Lack of time
Lugtenberg et al. 2015 [59–61]	Netherlands	To identify perceived barriers to using large-scale implemented CDS, covering multiple disease areas in primary care	**Condition of focus:** Range **Setting**: General Practice **Tool**: CDS for range of conditions, including CVD, asthma/COPD, diabetes, thyroid disorders, viral hepatitis, AF, subfertility, gastro protection and chronic renal failure - Embedded/linked with EMR: Yes - Interruptive alert: Yes - User-driven: GP - Risk score: No	Qualitative focus groups	Value of CDS in a primary care setting, CDS in an ideal world, experiences with using CDS with the example of NHGDoc, perceived advantages and disadvantages, and barriers to using them in practice	The system’s responsiveness was a problem, with the loading of alerts taking too long Many physicians mentioned that using CDS has a negative effect on patient communication during consultation and is considered a barrier to their use Discrepancy between a patient’s reason for visiting and the alert content was a reason not to use it Limited time during consultations made it difficult to use the CDS, as well as the additional work it requires	Perception **Impact on time**: Increase **Driving perception**: slow software, workload already heavy
Pannebakker et al. 2019 [62]	England	To understand GP and patient perspectives on the implementation and usefulness of the eCDS	**Condition of focus:** Cancer **Setting**: General Practice **Tool**: - Type: CDS for melanoma - Embedded/linked with EMR: Yes - Interruptive alert: No - User-driven: GP - Risk score: No	Qualitative interviews	GP and patient perspectives	Some reflected on how using CDS did not intrude in a consultation, and that it could help with saving time during or after a consultation	Perception **Impact on time:** Decrease **Driving perception:** efficiency, reduced time needed for data entry
Peiris et al. 2009 [63]	Australia	To develop a valid CDS tool that assists Australian GPs in global CVD risk management, and to preliminarily evaluate its acceptability to GPs as a point-of-care resource for both general and underserved populations	**Condition of focus:** Cardiovascular disease **Setting**: General Practice **Tool**: CDS for CVD risk management - Embedded/linked with EMR: No - Interruptive alert: Yes - User-driven: GP - Risk score: Yes	Mixed: quantitative survey + qualitative interviews	**Survey**: GP attitudes about the tool and management provided **Interviews**: general attitudes about the tool and its impact on the consultation; a review of specific tool outputs; recommendations for future tool development	Challenges: - Time pressures introduced by incorporating CVD risk management into routine care - Extra work seen in cases where the GP didn't expect CVD risk to be high, but these instances were few - Future automation of the tool (e.g. pre-population with data) seen as important Recommendations - ‘Too wordy' to read whilst with patient. For a consultation, 'you've got 15 min at most'	Perception **Impact on time:** Neither increase nor decrease **Driving perception**: work was increased only where risk was unexpectedly high, but this was not often
Rieckert et al. 2018 [64]	Germany	To examine how GPs experienced the PRIMA-eDS tool, how GPs adopted the recommendations provided by the CMR, and explore GPs’ ideas on future implementation	**Condition of focus:** Prescribing **Setting**: General Practice **Tool**: CDS to prevent inappropriate medication in older populations - Embedded/linked with EMR: No - Interruptive alert: No - User-driven: GP - Risk score: No	Qualitative interviews	1. Polypharmacy in everyday practice 2. Using the eCRF 3. General overview of the comprehensive medication review 4. Output of the CMR and how GPs responded to the recommendations 5. Implementation of the tool into daily practice routine	Entering patient data into the eCRF was time-consuming. After a period of familiarisation utilisation became easier and faster ‘For the first one I took 45 min I think and in the end it took me ten minutes’. (GP 14) Retrieving additional information provided by the tool was perceived as being too time-consuming	Perception **Impact on time:** Mixed views
Rickert et al. 2019 [65]	Germany	To examine how GPs experienced the PRIMA-eDS tool, how GPs adopted the recommendations provided by the CMR, and explore GPs’ ideas on future implementation	**Condition of focus:** Prescribing **Setting**: General Practice **Tool**: CDS to prevent inappropriate medication in older populations - Embedded/linked with EMR: No - Interruptive alert: No - User-driven: GP - Risk score: No	Quantitative survey	Use of and attitudes toward the CMR, its recommendations, and future use	Prerequisites for the future use of the PRIMA-eDS tool: Technical limitations were rated by 93% of GPs as important for future use of PRIMA-eDS, data security by 86%, and time requirement by 85% DISCUSSION: Previous research has shown that physicians ignored alerts when this was not the reason for the patients’ visit, as often there was not enough time to deal with both	Perception **Impact on time**: Unclear
Robertson et al. 2011 [66]	Australia	To determine GPs’ access to and use of electronic information sources and CDS for prescribing	**Condition of focus:** Prescribing **Setting:** General Practice **Tool**: CDS for prescribing - Embedded/linked with EMR: - Interruptive alert: - User-driven: GP - Risk score: No	Qualitative interviews	Electronic resources/CDS: - advantages and disadvantages of electronic over paper-based resources, - valued features of electronic decision support systems, - features of alerts and reminders (content, presentation and perceived usefulness), - support and training needs	GPs mentioned the pressures of a 10- to 15-min consultation, that their information needs were immediate at the point of care. GPs wanted relevant information presented concisely, easily searchable, integrated in the workflow and embedded in clinical software (the need to logon or go outside the main programme was seen as a burden and time waster)	Perception **Impact on time:** Unclear
Sperl-Hillen et al. 2018 [67]	USA	To evaluate whether the CDS intervention can improve 10-year CVD risk trajectory in patients in primary care setting	**Condition of focus:** Cardiovascular disease **Setting**: Primary Care **Tool**: assessment of CV risk - Embedded/linked with EMR: Yes - Interruptive alert: - User-driven: GP - Risk score: Yes	Quantitative: analysis of EMR + survey	**Primary:** CV risk values and clinical impact of the CDS system **Secondary:** Pre- and post (18 m) survey of Primary Care physicians: - Confidence and preparedness to address CV risk with patients - Satisfaction and perceptions with the CDS	PCPs reported that the CDS helped them to initiate discussions about CV risk (94%), improved CV risk factor control (98%), saved time when talking about CV risk with patients (93%), enabled efficient elicitation of patient treatment preferences (90%), supported shared decision making (95%), and influenced treatment recommendations (89%)	Perception **Impact on time:** Decrease **Driving perception:** saved time in conversations with patients
Sperl-Hillen et al. 2019 [68, 69]	USA	To evaluate improvements to clinical outcomes, impact on clinic workflow, use of CDS and satisfaction among clinicians	**Condition of focus:** Chronic disease **Setting**: Primary Care clinics **Tool:** CDS for chronic disease management & preventive care - Embedded/linked with EMR: Yes - Interruptive alert: - User-driven: GP - Risk score:	Quantitative: analysis of EMR, CDS useage data, survey of clinicians	Clinical outcomes Impact on clinic workflow CDS use rates Clinician satisfaction	93 percent reported it saved time when talking to patients about CV risk factor control	Perception **Impact on time:** Decrease **Driving perception:** saved time in conversations with patients
Sukums et al. 2015 [70, 71]	Sub-Saharan Africa	To describe health workers’ acceptance and use of the eCDS for maternal care in rural primary health care (PHC) facilities of Ghana and Tanzania and to identify factors affecting successful adoption of such a system	**Condition of focus**: Antenatal and intrapartum care **Setting**: Primary Health Care clinics **Tool**: - Type: CDS for antenatal and intrapartum care - Embedded/linked with EMR: Yes - Interruptive alert: No - User-driven: Clinician - Risk score: No	Mixed: quantitative survey + interviews	Perceived challenges affecting the eCDS use through a mid-term- and post- survey at 10 months (midterm) and 18 months (final) after implementation Interviews with the care providers were conducted to explore their views and experiences with the eCDS	Perceived increase in workload due to the eCDS use reported About one third of providers indicated a lack of time to use the eCDS Reasons given for these challenges: inadequate computer skills, inadequate staffing during busy periods Perceived workload also increased due to simultaneous manual and electronic documentation, which some providers felt to disrupt their work	Perception **Impact on time:** Increase **Driving perception:** stand-alone data entry, workload already heavy
Trafton et al. 2010 [72, 73]	USA	To evaluate the usability of ATHENA-OT, and to identify key needs of clinicians for both integrating the CDSS into their workflow and for opioid prescribing in general	**Condition of focus:** Prescribing **Setting**: Primary Care **Tool**: CDS for use of opioid therapy for chronic, non-cancer pain - Embedded/linked with EMR: - Interruptive alert: - User-driven: Clinician - Risk score: No	Mixed: quantitative and qualitative observations, survey, interviews and usage data	Usability of ATHENA-OT Key needs of clinicians	**Qualitative**: Many competing time constraints limit use of a CDS for OT. While the CDS streamlines and facilitates practices recommended in the CPG, they still require time to complete **Quantitative survey:** ATHENA-OT system was rated lowest on expectations that it would save time in visits **Provider Shadowing**: Clinic visits varied from 13 to 59 min and averaged 31 min. 10/35 visits involved the ATHENA-OT. In these 10 visits, the time ATHENA-OT was used ranged from 3 s to 10 min Clinicians appeared to have reasonable time to use the system. This contradicts clinicians’ self-reported lack of time during visits, reflecting either non-representativeness of the visits observed, or exaggeration of time constraints by clinicians	Perception and objective measure of time showed conflict **Impact on time:** mixed views
Trinkley et al. 2019 [74]	Canada	To describe current clinician perceptions regarding beneficial features of CDS for chronic medications in primary care	**Condition of focus:** Prescribing **Setting**: Primary Care **Tool**: CDS for prescribing chronic medications - Embedded/linked with EMR: - Interruptive alert: - User-driven: Clinician - Risk score: No	Qualitative focus groups	Beneficial CDS features for chronic medication management in primary care Participants' ideal CDS for chronic medications Potential unintended consequences of the CDS	Main beneficial features of alerts: (1) non-interruptive alerts; (2) clinically relevant and customisable support; (3) summarisation of pertinent clinical information and (4) improving workflow Alerts were “one more thing to get through” and a barrier to completing tasks. Clinicians reported ‘alert fatigue’, with an overwhelming number of alerts for ‘every patient’ While not universally endorsed, some indicated they liked one alert for lung cancer screening and found it helpful, because it interrupted workflow at the right time No consensus regarding best timing of an interruptive alert. Roughly equal numbers preferred the to alert at: (1) opening of an encounter; (2) ordering or reviewing a medications; (3) entering a diagnosis or (4) at the end of the encounter	Perception **Impact on time**: Unclear
Voruganti et al. 2015 [75]	Canada	To investigate current practices for assessing risk, awareness and use of risk assessment tools in primary care, and to assess PCPs’ perspectives regarding the usefulness, usability and feasibility of implementing computer-based health risk assessment tools into routine clinical practice	**Condition of focus:** Chronic disease **Setting**: Primary Care **Tool**: risk assessment for chronic diseases - Embedded/linked with EMR: - Interruptive alert: - User-driven: Clinician - Risk score:	Qualitative focus groups	PCPs' awareness of risk assessment tools, and views on their usefulness, usability and feasibility of routinely using them in clinical practice	Perceived benefits and shortcomings of tools: - beneficial for initiating discussion, engaging patients in risk discussions, and guiding decision-making by physicians and patients - concern about impact on workflow (“it might bring up a lot more other issues that they [patients] weren’t originally aware of and the discussion might actually… be less directed”) - some felt differently, that “it usually stops a lot of the meandering dialogue that you’d otherwise engage in” Expectations of an ideal risk assessment tool: - number of steps to complete a risk assessment should be minimised to a few clicks	Perception **Impact on time:** Mixed view
Walker et al. 2017 [76, 77]	Australia	To examine useability and acceptability of a prototype tool ‘CRISP’ (Colorectal cancer RISk Prediction tool), identify barriers and enablers to implementing CRISP in Australian general practice, and optimize the design of CRISP prior to an RCT	**Condition of focus:** Cancer **Setting**: General Practice **Tool**: risk assessment for colorectal cancer - Embedded/linked with EMR: - Interruptive alert: - User-driven: Clinician - Risk score:	Qualitative: simulated consultations + interviews	Acceptability, usability and implementation strategies were explored at an individual level (GP, PN and PM) and organizational level (the practice) using the four domains of NPT: - Coherence - Cognitive participation - Collective action - Reflexive monitoring	Collective action - GPs, PNs and PMs all agreed that lack of GP consultation time would limit the use of CRISP by GPs - Consensus that nurses have the capacity, time and expertise to complete the risk assessment as part of routine preventive health consultations - Opinions about who would take responsibility for the final decision about screening advice was split between GPs and PNs; many GPs feared missing a diagnosis	Perception **Impact on time:** Increase **Driving perception**: lack of time
Zazove et al. 2017 [78]	USA	To develop a model electronic alert that integrates into system 1 thinking (thinking that is fast and intuitive, often occurring without much conscious thought), which family medicine clinicians would use to improve identification of individuals at risk for HL	**Condition of focus:** Hearing loss **Setting**: Family Medicine clinic **Tool**: risk assessment for hearing loss - Embedded/linked with EMR: - Interruptive alert: - User-driven: Clinician - Risk score:	Mixed: cognitive task analysis interviews	How often various issues were identified, the root causes of use and non-use of the electronic alert, sample quotes highlighting major issues, and any potential solutions mentioned	Time pressure with electronic prompts: - Clinicians felt visits were already overloaded, limiting their ability to handle additional alerts. Addressing all recommendations for complex patients requires more than the typical 15-min office visit - Alerts intrude on the doctor-patient relationship, since they rarely address the primary reason for the visit, and the added workload contributes to clinician stress due to falling further behind in the schedule Hearing loss is not easily addressable: - the “time pressure” was due to clinicians being uncomfortable and having to take time to think about how to address HL (ie, being forced into slow and effortful system 2 thinking), which they did not have to do with other conditions	Perception **Impact on time:** Increase **Driving perception:** workload already heavy
Murdoch et al. 2015 [79, 80]	UK	To use conversation analysis to assess the interactional workability of using CDS for telephone triage	**Condition of focus**: Same-day appointment requests (range of conditions) **Setting**: General Practice **Tool**: CDS to guide nurse-led telephone triage - Embedded/linked with EMR: Yes - Interruptive alert: No, guides triage call - User-driven: Nurse - Risk score: No	Qualitative: conversation analysis	1. Structuring of patient's problem 2. making sense of/managing pt's symptoms in the CDS 3. where pts' experience misaligned with CDS requirements 4. nurse accountability within CDS 5.Consequences of not using CDS on Qs and answers	Use of CDS impacted call 'trajectory' and caused disruptions 'interactional workability' of using the CDS for telephone triage Eg. Operational problems such as mistyping a symptom or condition led to a 'prolonged pause' while nurse attempted to correct/search around for another term and explain the delay to the patient	Objective measure of time of whole consultations but timings data not reported **Impact on time:** unclear
Jetelina et al. 2018 [81]	USA	Proof of concept study: before and after implementation of e-tool	**Condition of focus:** Behavioural Health **Setting**: Primary care clinics **Tool**: suite of e-tools for Behavioural Health Clinicians - Embedded/linked with EMR: Yes - Interruptive alert: No - User-driven: Clinician - Risk score: No	Mixed: quantitative surveys + qualitative interviews and observations	Clinical outcomes and patient experience Acceptability of the e-tools Factors influencing implementation	Acceptability: - Tool was acceptable and easy to use - Tool added 1 to 2 min to the initial visit but time during follow-up visits by automatically populating the history of the presenting illness and patient instructions at subsequent visits	Perception **Impact on time:** Decrease **Driving perception:** efficiency, reduced time needed for data entry
McGinn et al. 2013 [82]	USA	To examine the effect of the tools on diagnostic and treatment patterns and to assess adoption of the tool for each condition	**Condition of focus:** Upper Respiratory Tract Infections **Setting**: Primary care clinics **Tool**: CDS with clinical prediction rules for 2 URTIs - Embedded/linked with EMR: Yes - Interruptive alert: Yes - User-driven: Clinician - Risk score: Yes	Quantitative: analysis of EMR and usage data	Usage data: number of visits involving: - tool being opened once triggered - calculator being completed - viewing of recommendations and - following of recommendations	Time not measured or commented on Regarding diagnostic/treatment/management patterns, no significant differences between arms in proportions of visits resulting in patient returning to ED/Outpatient clinic for follow-up High adoption rates reported	Objective workload measure of follow-up visits **Impact on workload:** neither increase nor decrease
Litvin et al. 2016 [83]	USA	To assess impact on CKD clinical quality measures and facilitators/barriers to use of tools	**Condition of focus:** Chronic Kidney Disease **Setting**: Primary care clinics **Tools:** CDS tools for CKD, including risk assessment - Embedded/linked with EMR: Yes - Interruptive alert: Yes - User-driven: Clinician - Risk score: Yes	Mixed: quantitative analysis of EMR data + qualitative observations and interviews	**Quantitative**: CKD clinical quality measures at baseline & 2 years **Qualitative**: Barriers/facilitators to using tools	Barriers included mention by some staff that CDS tools required 'extra clicks' and additional steps outside of existing workflow	Perception **Impact on time**: Increase **Driving perception**: workflow disruption
Linder et al. 2009 [84]	USA	To assess effect of CDS on antibiotic prescribing rates for ARI visits	**Condition of focus:** Acute Respiratory Infections **Setting**: Primary care clinics **Tool**: CDS for ARIs to help reduce inappropriate prescribing - Embedded/linked with EMR: Yes - Interruptive alert: Yes - User-driven: Clinician - Risk score: Yes	Quantitative: analysis of EMR data	**Primary:** Rate of antibiotic prescribing for ARI visits **Secondary**: 30-day re-visit rates attributable to ARIs	Recorded duration of Smart Form use (assume mean) 8.1 m, sd 5.8 m. However, whole visit length not captured/reported and not compared with Usual Care Re-visit rate attributable to ARIs 8% in intervention and 9% in control but not remarked upon in Discussion or quantified as significant or not significant Remarked in discussion that to be effective, CDS must fit as seamlessly as possible into existing workflow and allow clinicians to manage unanticipated interruptions	Objective measure of time to use tool and revisit rates **Impact on time:** Unclear Revisit rates not remarked upon
Ranta 2013 [85]	New Zealand	To assess feasibility of introducing risk tool to help timely management of TIAs	**Condition of focus:** TIA & Stroke **Setting**: General Practice **Tool**: CDS for TIA & Stroke risk - Embedded/linked with EMR: Yes - Interruptive alert: No - User-driven: GP - Risk score: Yes	Quantitative: analysis of usage data + survey	Usage of the tool and advice rendered/actions taken by GPs Post-pilot satisfaction of GP users	GP survey interviews reported 'no major concerns' regarding the time required to enter data (Methods report this to be 3-5 min per TIA patient) Time not formally measured and no indication given of whether time is added to the consultation (perhaps 'acceptably')	Perception **Impact on time**: Unclear
Price et al. 2017 [86, 87]	Canada	To examine how 40 STOPP rules could be implemented as alerts into practice and the impact on prescribing	**Condition of focus:** Prescribing **Setting**: Family Practices **Tool**: Screening tool of older people's prescriptions (STOPP) - Embedded/linked with EMR: Yes - Interruptive alert: Yes - User-driven: Physicians - Risk score: No	RCT with mixed methods: quantitative analysis of EMR data + interviews	**Quantitative**: change in rate of potentially inappropriate prescriptions (PIPs) between arms **Qualitative**: views on barriers and facilitators of implementation	Qualitative interviews - 'workflow' cited as a barrier to implementation, along with 'location' of alert on-screen - Workflow not expressed in terms of time (increase or decrease) or sequence of activity and not quantified or measured as time	Perception **Impact on time**: Unclear
Wan et al. 2010 [88]	Australia	To explore GPs and patients' views of implementing CVAR assessment, including issues regarding identifying patients at risk and the timing and context for assessment	**Condition of focus:** Cardiovascular disease **Setting**: General Practice **Tool**: Range of electronic and paper-based tools for cardiovascular absolute risk assessment - Embedded/linked with EMR: Unclear - Interruptive alert: Unclear - User-driven: Unclear - Risk score: Yes	Qualitative interviews	Views of barriers and facilitators of implementing CVAR assessment	No specific e-tool examined and time not mentioned, but a general comment in Discussion that GP workload pressure is an important barrier to increasing preventive activity in general	GP workload pressure **Impact on time/workload**: no conclusion
Hor et al. 2010 [89]	Ireland	To assess prevalence and use of EMR and any form of CDS for prescribing and to explore perceived benefits of future introduction of CDS-eP, barriers to implementation and presumptive responses to prescribing alerts	**Condition of focus:** Prescribing **Setting**: General Practice **Tool**: Hypothetical CDS for e-prescribing - Embedded/linked with EMR: Yes - Interruptive alert: Yes - User-driven: Unclear - Risk score: No	Quantitative: survey + free text	Prevalence and use of EMR and any form of CDS for prescribing GPs' perceived benefits of future introduction of CDS-eP, barriers to implementation and presumptive responses to prescribing alerts	Time not measured but mentioned in GPs' general comments: hypersensitive interruptive alerts whilst prescribing and causing a delay to prescribing by e.g. 20 s would be frustrating	Perception **Impact on time:** Increase **Driving perception**: workload already heavy
Troeung et al. 2016 [90]	Australia	To develop and evaluate performance of the e-screening tool against practice EMR data	**Condition of focus:** Familial hypercholesterolaemia **Setting**: General Practice **Tool**: screening tool to identify patients with familial hypercholesterolaemia - Embedded/linked with EMR: Yes - Interruptive alert: No - User-driven: GP - Risk score: Yes	Quantitative: analysis of EMR and tool data	Performance of the e-screening tool	Time reported (10 min) to run the e-screening search, not consultation length	Objective measure of time to use tool **Impact on time**: Unclear
Jimbo et al. 2013 [91]	USA	To identify PCPs' perceived barriers and facilitators to implementation	**Condition of focus:** Cancer **Setting**: General Practice **Tool**: CDS for patients regarding their colorectal cancer screening preferences, linked to computerised reminder alerts for clinicians - Embedded/linked with EMR: Yes - Interruptive alert: Yes - User-driven: Patient - Risk score: No	Qualitative focus groups	Barriers and facilitators to implementation	Clinicians (majority) identified principal barriers to patients' access to colorectal cancer screening and patients' concerns regarding costs, but also time constraints during visits to discuss the screening options (e.g. could mean a 5–10 min conversation)—the web-based tool completed by patients prior to their visit could potentially save time at the visit Some concerns over how clinician reminder alerts would fit into the usual visit workflow, but not expanded upon	Perception **Impact on time**: mixed view
Akanuwe et al. 2020 [92]	UK	To explore the views of service users and primary care practitioners on how best to communicate cancer risk information when using QCancer, a cancer risk assessment tool, with symptomatic individuals in primary care consultations to enable them be involved in decisions on referral and cancer investigations	**Condition of focus:** Cancer **Setting**: General Practice **Tool**: Qcancer risk assessment tool - Embedded/linked with EMR: Yes - Interruptive alert: Yes - User-driven: GP - Risk score: Yes	Qualitative interviews and focus groups	Personalising risk information Informing and involving patients Being open and honest Providing time for listening, explaining and reassuring in the context of a professional approach	‘Talking about risk is quite difficult’ - GP may be reluctant to inform the patient about cancer risk when they themselves were uncertain about the risk calculated or how to communicate this **Patients**: - GPs should take time to talk to patients to gain their confidence and show they care: 'You wouldn't want to feel that you've been rushed’ - ‘GPs would need more time to use the tools in consultations' **GPs**: GPs expressed the need to provide more time to provide explanations to patients	Perception **Impact on time:** Increase **Driving perception:** time needed to use the tools, convey information and show caring
Bangash et al. 2020 [93]	USA	To develop a CDS tool for Familial Hypercholesterolemia based on physician feedback from qualitative interviews, usability testing and an implementation survey	**Condition of focus**: Familial Hypercholesterolemia **Setting**: Primary Care **Tool**: CDS for FH - Embedded/linked with EMR: Yes - Interruptive alert: Yes - User-driven: GP - Risk score: Yes	Mixed: Qualitative interviews, usability testing + survey		The most common barrier is the increasing cognitive burden on providers due to EMR complexity and limited time during clinical encounters Most physicians are receptive towards CDS. The only survey item where the majority of the physicians gave either a neutral response or disagreed was regarding the CDS tool ‘not’ increasing time spent with a patient This response reiterated the need for CDS to be designed to increase e ciency and not add to provider burden	Perception **Impact on time:** Increase **Driving perception:** workflow disruption, limited time during consultations
Bradley et al. 2021 [94]	UK	To synthesise qualitative data of GPs’ attitudes to and experience with a range of CDs to gain better understanding of the factors shaping their implementation and use	**Condition of focus:** Cancer **Setting**: Primary Care **Tool**: Range of CDS tools for cancer - Embedded/linked with EMR: Range - Interruptive alert: Range - User-driven: Range - Risk score: Yes	Systematic review—qualitative synthesis	**Impact of CDS on role of GP** - communicating risk - collaboration with secondary care/guidelines - nature of training provided **Elements determining GPs' use** - clinical acumen v protocol - medicolegal issues **Impact on GPs work** - increasing awareness of cancer - prompt fatigue - impact of IT integration (time) - time as a resource **GPs' reflections** - unintended consequences - investigation and referral patterns - 'think cancer'	**Prompt fatigue** - Prompt fatigue was mentioned by several studies. The interruptions impacted on the flow of the consultation The prompts were regarded, in some studies, as making work more difficult; another commented on the usefulness of prompts for future consultations **Impact of IT integration** - ‘It was not easy accessing the tools during patient consultation’ **Time as a resource** - Recognition of the benefits of using a CDS was essential to justify the additional time required for its use. This impacts on consultation, time required to train users and the additional effort to continue using the CCDT. Time is at a premium in general practice in the UK: the pressures of the 10 min appointment, to keep up to date and to attend training	Perception **Impact on time**: Mixed views **Driving perception**: time consuming to complete, limited time during consultations
Breitbart et al. 2020 [95]	Germany	To assess how CDS vs standard consultations affect patient satisfaction, diagnostic accuracy and length of consultations	**Condition of focus:** Skin conditions **Setting**: General Practice **Tool**: Visual CDS for dermatology consultations - Embedded/linked with EMR: No - Interruptive alert: No - User-driven: GP - Risk score: No	Randomised feasibility study	Patient experience and satisfaction Diagnostic accuracy Consultation length	The median duration of the consultations using the CDS was 10 min, similar to that in the standard arm In the CDSS arm, overall patient satisfaction correlated negatively with increased duration of consultation (*P* = 0.02) Across both arms younger patients (20-40y) were more bothered about consultation length relative to the older patients Overall, the CDSS increased aspects of patient satisfaction, improved diagnostic accuracy **without influencing the duration of the consultation**	Objective measure of time of whole consultations **Impact on time:** neither increase nor decrease
Byrne et al. 2015 [96]	Ireland	To benchmark the awareness and use of Risk Assessment tools and CVD prevention guidelines along with barriers to their use among a sample of Irish GPs	**Condition of focus:** CVD **Setting** [16, 97]: General Practice **Tool**: CVD risk assessment tools - Embedded/linked with EMR: - Interruptive alert: - User-driven: GP - Risk score:	Cross-sectional survey of GPs	Demography Risk assessment CVD guideline use Perception of barriers to use of Risk Assessment tools and CVD guidelines	Main three barriers to use of Risk Assessment tools: 1) patients focused on a single risk factor and not global picture (32.9%) 2) **time constraints** (30.6%) 3) not being used to using a risk calculator (18.4%) Barriers to implementation of CVD prevention guidelines: - lack of remuneration (40.8%) - too many CVD guidelines (38.9%) - **time constraints** (35.7%)	Perception **Impact on time:** Increase **Driving perception:** limited time during consultations
Caturegli et al. 2020 [98]	South Africa	To pilot a prescribing tool	**Condition of focus:** Tuberculosis **Setting**: Primary Care clinics **Tool**: prescribing tool for TB preventive therapy - Embedded/linked with EMR: No - Interruptive alert: No - User-driven: Clinician - Risk score: No	Mixed methods	Prescribing rates Perceived barriers to prescribing Workload Stock-outs Prescription guidelines Intervention impact Cognitive load Documentation	**Workload** - Reduces the writing one has to do **Documentation** - According to five of eight providers, time spent documenting medications and contraindications was reduced with the tool	Perception **Impact on time:** Decrease **Driving perception:** reduced time spent documenting medications and reduced cognitive load
Chadwick et al. 2017 [99]	UK	To evaluate feasibility and acceptability of a prototype application of a risk stratification algorithm incorporated into a CPOE and triggering a prompt to offer an HIV test when the healthcare worker is ordering other tests	**Condition of focus:** HIV **Setting**: Hospitals and general practices **Tool**: HIV testing prompt within a Computerised Physician Order Entry system - Embedded/linked with EMR: Yes - Interruptive alert: Yes - User-driven: Clinician - Risk score: No	Qualitative: Interviews and focus groups	Frequency and appropriateness of the prompt The prompt in the context of the consultation Reactions of patients to the prompt Impact of the prompt on HIV testing	Frequency and appropriateness of the prompt - Little evidence of “prompt fatigue”. This particular prompt, compared with other prompts, was considered simple to understand and easy to manage The prompt in the context of the consultation - Most discussed blood tests and submitted an order with the patient present. Some GPs ordered the test after the patient had left and were faced with the dilemma of whether to bring the patient back to discuss HIV testing - Many hospital-based and general practice HCWs felt the prompt was too late in the ordering process and disrupts the consultation, potentially opening up a new topic, causing irritation	Perception **Impact on time:** Mixed views **Driving perception:** Either: - no mention of time - prompt causes test to be ordered after consultation - disruptive if prompt comes too late in the ordering process - potentially opens up a new topic right at the end of the consultation
Chadwick et al. 2021 [100, 101]	UK	To evaluate a prototype application designed to prompt in real-time, BBV testing in previously untested higher risk individuals attending primary care	**Condition of focus**: Blood-borne viruses **Setting**: General practice **Tool**: CDS to identify patients at risk of blood-borne viruses - Embedded/linked with EMR: Yes - Interruptive alert: Yes - User-driven: Clinician - Risk score: Yes	Prospective cohort study	Number of 'hard prompts' and clinicians' responses BBV tests ordered Survey of GPs	Clinicians’ perceptions of the prompt system were positive with average additional time required for BBV test discussion in consultations **estimated at 2 min** Nineteen percent of clinicians reported having to make **an additional appointment after a BBV test prompt because of insufficient time during a consultation** and 15% had to make an additional appointment to discuss test results Free-text answers stressed the lack of time available **Median additional consultation time varied from 0.25 min when the clinician ignored the prompt to 2 min when the prompt was accepted or declined**	Perception **Impact on time:** Increase **Driving perception:** limited time during consultations
Chima et al. 2019 [102]	UK	To summarise existing evidence on the effects of eCDSTs on decision making for cancer diagnosis in primary care, and determine factors that influence their successful implementation	**Condition of focus:** Cancer **Setting**: General practice **Tool**: Range of CDS tools to support cancer diagnosis - Embedded/linked with EMR: Range - Interruptive alert: Range - User-driven: Range - Risk score: Range	Systematic review	Appropriateness of care (*n* = 5); Diagnostic accuracy (*n* = 1); Time to diagnosis (*n* = 1); Cost-effectiveness (*n* = 1); Process measures (*n* = 1); and Qualitative (*n* = 4)	Introducing the tool disrupted the consultation, with GPs reporting feeling a loss of control Additional tasks and time pressures impacted clinical flow For eCDSTs designed for use during consultation, there were challenges due to disruption of the usual workflow and the generation of additional tasks in an already-busy appointment	Perception **Impact on time:** Increase **Driving perception:** limited time during consultations, disruption, loss of control, additional tasks and time pressures, impact on clinical flow
Dobler et al. 2019 [103]	USA	To determine clinician outcomes in RCTs of encounter decision aids for Shared Decision Making	**Condition of focus:** Range **Setting**: General practice **Tool**: Range of CDS tools to support shared decision making of screening/treatment options in consultations - Embedded/linked with EMR: Range - Interruptive alert: Range - User-driven: Range - Risk score: Range	Systematic review	Clinician satisfaction - clinical encounter - decision-making process - the decision aid - the decision made Efficiency - consultation length Personal and professional well-being - mood and burnout - satisfaction with the practice of clinical care	**Clinician satisfaction** - Communication was enhanced by providing visual representations of choices, reduced clinicians’ burden to produce accurate representations, giving clinicians more time to engage in meaningful discussions with patients - Clinicians' concerns included that decision aids would add time to their clinics if they were not simple **Efficiency** - One study showed that 77% of clinicians in the decision aid group thought the decision aid was not disruptive and even potentially beneficial, 15% found it neither disruptive nor beneficial and 8% found it potentially disruptive - 9/13 studies measuring consultation length found no significant difference in time between intervention and control groups. Three studies reported a longer and one study a shorter consultation time in the decision aid group - One study evaluating why some of the clinicians in the intervention group did not use the decision aid in consultations, showed that clinicians’ perception that they do not have enough time was the main reason for not using it. Length of consultation was not measured, so it is unclear if using the decision aid did prolong the consultation time	Objective measure of time of whole consultations **Impact on time**: neither increase nor decrease **Driving perception:** limited time during consultations, perception that it will add time
Fiks et al. 2015 [104]	USA	To characterize patterns of adoption of the CDS system, assess the impact of performance feedback on CDS adoption by primary care clinicians, and measure the impact of CDS use on guideline adherence	**Condition of focus:** Otitis Media in children **Setting**: Primary care **Tool**: CDS for Otitis Media - Embedded/linked with EMR: Yes - Interruptive alert: Yes - User-driven: Clinician - Risk score: No		Adoption of CDS Impact of feedback on adoption - use of documentation or order entry panels Adherence to guidelines Visit-level covariates: - visit type - type of OM	Clinicians concerned regarding the number of “clicks” needed to use the system, which was perceived as inefficient Clinician enthusiasm for the tool was decreased because of the change in workflow that was required, especially for visits with multiple problems Clinicians ignored the tool at 80 percent of eligible visits. Two percent of clinicians never used the CDS, and 11 percent used the tool during a trial period but not again	Perception **Impact on time:** Increase **Driving perception**: inefficient to use and causes changes in workflow
Ford et al. 2021 [105]	UK	To support and optimise the design of future CDSs by identifying factors that influence how or why GPs use these tools, looking specifically into aspects of CDSs they find useful and problematic, both individually and in the wider context of their practice	**Condition of focus:** Dementia **Setting**: General Practice **Tool**: Hypothetical CDS for dementia risk prediction - Embedded/linked with EMR: Range - Interruptive alert: Range - User-driven: Range - Risk score: Range	Qualitative interviews	Trust in individual CDS Usability of CDS in consultation context Usability of CDS in broader practice context	**Intrusiveness** Perceived as unhelpful where CDS raised an issue which the GP felt to be unimportant within that particular consultation; where the alert does not relate to a topic of importance for either GP or patient, it may be perceived as undermining of the GP’s professional expertise - Self-population of CDS fields using previously- recorded data (e.g. in QRISK) was viewed as a benefit which reduced intrusiveness and time pressures **Alert fatigue** - Negative impact on patient-doctor rapport: “It really is like an interruption”, “no GP wants somebody to just burst in with the door opening or the phone ringing. In the same vein no GP really wants a big thing to just pop up on the screen that they didn’t call up.” - proliferation of alerts led to participants becoming desensitised to alerts, which could cause GPs to miss important safety alerts	Perception **Impact on time:** No conclusion **Driving perception:** limited time in consultations, workload already heavy
Henshall et al. 2017 [106]	UK	To explore the views of clinicians, patients and carers.on feasibility and acceptability	**Condition of focus:** Psychiatric disorders (schizophrenia) **Setting**: General Practice **Tool**: Cloud-based CDS algorithm providing information on interventions - Embedded/linked with EMR: Unclear - Interruptive alert: Range - User-driven: Clinician - Risk score: No	Qualitative focus groups	Applications in clinical practice Communication Conflicting priorities Record keeping and data management	**Applications in clinical practice** - the tool did not reflect the complex clinical assessment process, being unable to capture detailed information about patient characteristics, time pressures, anxiety, influence of carers, clinician experience and organisational factors - “When we see someone… it changes the conversation…Depending on how much time you have” **Communication** - some clinicians and patients/carers highlighted that receiving too much information might cause unnecessary worry and result in clinicians spending considerable time reassuring patients - risk of entering into a ‘minefield of discussion’	Perception **Impact on time:** Mixed views **Driving perception**: limited time in consultations, discussion will add time
Holmstrom et al. 2019 [107]	Sweden	To describe factors affecting the use of a decision support tool and experiences among Telephone Nurses in Swedish primary health care	**Condition of focus**: Telephone nursing (range of conditions) **Setting**: Primary Care **Tool**: CDS providing guidelines/information and documentation in patient records - Embedded/linked with EMR: Yes - Interruptive alert: No, guides telephone call - User-driven: Nurse - Risk score: No	Qualitative: observations and interviews	Factors that decrease or cause deviation from CDSS Positive factors CDS complicates work	Long working experience, time pressure, lack of training, and non-native callers decreased CDS use Because of time constraints, the TN sometimes chose to rely on their own professional knowledge instead of the CDS	Perception **Impact on time:** Increase **Driving perception:** limited time in consultations, reading text in the tool takes time
Kostopoulou et al. 2017 [108, 109]	UK	To measure the prototype’s effectiveness, usability, and potential impact on the consultation and patient satisfaction	**Condition of focus:** Generic **Setting**: General Practice **Tool**: CDS for diagnostic support for a range of conditions - Embedded/linked with EMR: Yes - Interruptive alert: Yes - User-driven: Clinician - Risk score: No	Simulated patient study	Vision IT system recorded length of time patient record was open GP and patient survey(s), including length of consultation	Mean length of baseline consultatio*n* = 13.73 min (2.96 SD) Mean length of CDS consultatio*n* = 14.42 (5.28 SD) Neither the number of investigations nor the length of consultation differed significantly between the baseline and CDS sessions Patient satisfaction re consultation length similar at baseline and CDS consultations	Objective measure of time of whole consultations **Impact on time:** neither increase nor decrease
Laka et al. 2021 [110]	Australia	To identify the different individual, organisational and system level factors that influence the adoption and use of CDS	**Condition of focus:** Antibiotic management **Setting**: Hospital and general practices **Tool**: Range of CDS for antibiotic management - Embedded/linked with EMR: Range - Interruptive alert: Range - User-driven: Range - Risk score: Range	Quantitative survey	Survey - Perceived benefit - Perceived barriers - Perceived facilitators Free-text comments - Lack of flexibility - Information overload - Information accuracy	**Perceived barriers** - Clinicians in primary care more likely than those in hospital to believe that factors such as time limitation restrict the use of CDS **Information overload:** - Time and workload pressures make it difficult for clinicians to distinguish important information from irrelevant data	Perception **Impact on time:** Increase **Driving perception:** limited time in consultations
Lemke et al. 2020 [111, 112]	USA	To assess PCPs’ views of the tool and genomics-based CDS in clinical practice	**Condition of focus:** Geonomics (family health history screening) **Setting**: Primary Care **Tool**: Geonomics-based CDS to identify patients at risk - Embedded/linked with EMR: Yes - Interruptive alert: No - User-driven: Patient completes tools, which alerts clinician based on answers - Risk score: No	Qualitative interviews	Benefits to clinical care Challenges in practice CDS issues Physician-recommended solutions	**Time**: - Adding another topic to the patient’s annual visit, such as the alert recommendations, was difficult because of time constraints due to discussing other recommended screens and agenda items. Discussing the family history tool findings sometimes created difficulties in time management. “Because [physicians are] rushed and overburdened, if they know it’s going to take a lot of time, they’re going to ignore it.” **Workflow:** Many clinicians reported having tight schedules and felt that adding another component to the visit, would tax existing processes	Perception **Impact on time**: Increase **Driving perception**: limited time in consultations, adds burden
Li et al. 2012 [113]	USA	To assess how providers interact with the CDS while interviewing a simulated patient and to identify barriers to use prior to the implementation of a randomized controlled trial	**Condition of focus:** Upper Respiratory Tract Infections **Setting**: Primary Care **Tool**: CDS providing clinical prediction rules for Strep or Pneumonia - Embedded/linked with EMR: Yes - Interruptive alert: Yes - User-driven: Clinician - Risk score: Yes	Usability testing and simulated patient study	Usability issues: - Usability - Navigation - Content - Workflow	Overall perception of the CDS had a positive-to-negative commentary ratio of 0.86 favouring the negative; the categories of ‘Navigation’ ‘Workflow’ were associated with the largest volume of negative comments **Average encounter duration: 2.03 min** (5.11–18.35 min) In 71% of cases (*n* = 17) the CDS was triggered after an average of 51% of the visit had elapsed. **Clinicians spent on average 12.2% of encounter time using the CDS** Timing of trigger- (1) Visits where CDS accessed at the beginning of the visit lasted on average 13:26 min (2) Visits where CDS accessed at the end of the visit lasted on average 5:09 min	Objective measure of time of whole consultations **Impact on time:** no conclusion as no comparison with control
Lo et al. 2018 [114]	Australia	To assess the usability and acceptability of the iPrevent prototype	**Condition of focus:** Cancer **Setting**: Primary Care **Tool**: Breast cancer risk assessment, providing tailored risk management information - Embedded/linked with EMR: Yes - Interruptive alert: No, guides visit - User-driven: Clinician - Risk score: Yes	Quantitative usability testing Piloting using both simulated and real patients	Usability Acceptability Risk perception Knowledge Time spent completing tool Consultation time	The median time taken for clinician consultations in which iPrevent data were discussed was 20 (range 5–45) minutes Majority of clinicians felt the length of the tool was 'too long'	Perception and objective measure of time **Impact on time:** mixed picture **Driving perception**: length of time to complete tool too long
Margham et al. 2018 [115]	UK	To evaluate the impact of the electronic trigger tool, including acceptability to clinicians, ease of use, and rates of finding patient safety events	**Condition of focus:** Range **Setting**: Primary Care **Tool**: Trigger tools to identify patients at risk of safety-related incidents, including diagnostics, medication and communication, across a range of conditions - Embedded/linked with EMR: Yes - Interruptive alert: No, audit tool - User-driven: Clinician - Risk score: Yes	Mixed methods	**Quantitative** - numbers of patients identified and reviewed - rate of identification of patient safety events **Qualitative** - barriers and benefits to implementation, - ease of use - value of the trigger tool in the context of a busy GP surgery	GPs all expressed concern that the tool might identify too many patients at risk of harm, place further demands on GP time, and require additional resources to manage properly - ‘Heart said “good idea”. Head said “hope it doesn’t significantly increase my workload”!’	Perception **Impact on time:** Increase **Driving perception:** limited time in consultations, workload already heavy
North et al. 2016 [116]	USA	To examine clinician time involved in risk calculation and decision making. This was done in a setting to estimate the minimum time it might take a provider at the point of care	**Condition of focus:** Cardiovascular disease, Atrial Fibrilation, Diabetes and Heart Failure **Setting**: Primary Care **Tool**: CVD risk assessment (within Ask Mayo Expert) - Embedded/linked with EMR: Yes - Interruptive alert: Unclear - User-driven: Clinician - Risk score: Yes	Case scenarios Morae® Recorder software used to collect timing and usage	Risk calculation time Combined risk calculation and clinical decision making time	AF CHADSVASC risk calculation 36 s (9 s) AF total management time 85 s (18 s) Lipids AHA-ASCVD risk calculation 45 s (12 s) Lipids total management time 110 s (32 s) HF SHFM risk calculation 171 s (42 s) HF total management time 347 s (89 s)	Objective measure of time to use tool **Impact on time**: authors concluded that time spent on risk calculation can be reduced by using automated algorithms
Olakotan et al. 2021 [117]	Malaysia	To identify factors affecting the appropriateness of CDSS alerts in supporting clinical workflow based on the proposed evaluation measures for CDSS alert	**Condition of focus:** Range **Setting**: Primary Care **Tool**: Range of CDS tools generating alerts to support clinical workflow - Embedded/linked with EMR: Yes - Interruptive alert: Yes - User-driven: Clinician - Risk score: No	Systematic review	Technology factors Human factors Organisational factors Process factors	**Technology:** 5 studies reported that alert overload increases the mental workload of clinicians **Human** - 11 studies showed that EMR-embedded asynchronous alerts increase clinicians’ workload - Providing clinicians with protected time to respond to alerts reduces alert-related workload and improves patient safety - Other instances of workload involve clinicians documenting clinical data into EMR before an alert can be triggered and selecting reasons for bypassing alerts	Perception and objective measure of workload **Impact on workload**: Increase **Driving perception**: alert overload increases mental workload, physical and cognitive weariness, no time to respond to alerts, time needed for documenting clinical data and selecting reasons for bypassing alerts
Porat et al. 2017 [108, 109]	UK	To identify facilitators and barriers to future DSS adoption	**Condition of focus:** Range **Setting**: General Practice **Tool**: CDS tools to support diagnosis - Embedded/linked with EMR: Yes - Interruptive alert: Yes - User-driven: Clinician - Risk score: No	Mixed: Qualitative interviews with GPs + quantitative survey of patients	Perception of GPs Satisfaction of patients	**Impact on consultation style and GP-pt interaction**: - ‘You need to get used to it…I do my consultations in a different way, but it works quite quick’ - Eight GPs (23%) were concerned that typing during the consultation would interfere with doctor-patient communication: “I normally chat and look at the patients, it throws my normal thing.” “I usually don’t code during the consultation, less contact with patient, my style is to listen for a long time.” **Time concerns** - Thirteen GPs (38%) felt consultation took longer with the tool than without. Without the tool, using only the EMR, GPs wrote mainly free text, which they perceived to be faster: “It will be hard to use it in a 10-min. consultation.” - Average consultation time did not significantly differ between baseline and CDS sessions - The GPs who expressed concerns about time took longer when using the CDS (mean time 15.45 min) than in the baseline session (mean time 13.53 min), paired samples t-test: 2.13, df = 12, *P* = 0.055, but this was not the case for the whole GP sample - Despite concerns about time, GPs believed that they could become better using the CDS	Perception and objective measure of time **Impact on time**: neither increase nor decrease **Driving perception**: searching and selecting symptom codes takes longer than typing free text in patient notes as usual, limited time in consultations
Richardson et al. 2017 [118]	USA	To understand the determinants of usability of two CDS tools for lessons and themes that could be generalizable to all forms of CDS	**Condition of focus:** Upper Respiratory Tract Infections **Setting**: General Practice **Tool**: CDS for antibiotic ordering for URTIs, using a clinical prediction rule for risk assessment of either A Streptococcus, pharyngitis or pneumonia - Embedded/linked with EMR: Yes - Interruptive alert: Yes - User-driven: Clinician - Risk score: Yes	Observational study (1) Think aloud testing using written case scenario (2) Near live testing using simulated patients	Visibility Workflow Content Understandability Navigation	Duration of each think aloud/near live scenario: 25—45 min - the automatic order set, automatically generated documentation and communication with nurses and patients decreased workload and saved time Passive alerts triggered at the time of decision making allow clinicians to use tools without disturbing their natural workflow “I much prefer to have stuff in the background that doesn’t force me to have hard stops… There may be a whole series of other things I’m dealing with."	Perception **Impact on time**: Decrease **Driving perception**: can be used in such a way as to fit in with workflow and decision making
Richardson et al. 2019 [119]	USA	To further understand the barriers and facilitators of meaningful CDS usage within a real clinical context	**Condition of focus**: Upper Respiratory Tract Infections **Setting**: General Practice **Tool**: CDS for antibiotic ordering for URTIs, using a clinical prediction rule for risk assessment of either A Streptococcus, pharyngitis or pneumonia - Embedded/linked with EMR: Yes - Interruptive alert: Yes - User-driven: Clinician - Risk score: Yes	Qualitative observational study	Tool Interruptions Workflow Tool Applicability Patient-Tool interaction Provider-Computer-Patient Interaction Ease of Use Missed Opportunities	- Of 6 patient encounters, 5 were acute or follow-up visits that lasted about 15 min each, 1 was a complete physical exam that was about 30 min in length - Clinicians spent 0%-3% of visit time listening to the patient without engaging with the computer - Clinicians completed the tool quickly; however, during half of the visits, hard stops and fixed elements in the tool created barriers. Clinicians spent about 1 min of the visit time completing the CDS tool	Objective measure (estimate) of time of whole consultations **Impact on time**: neither increase nor decrease
Rubin et al. 2021 [16]	UK	To establish the tool's acceptability and collect relevant data to inform the design of a subsequent definitive trial	**Condition of focus:** Cancer **Setting**: General Practice **Tool**: CDS risk assessment tool for oesophageal-gastric cancer - Embedded/linked with EMR: No - Interruptive alert: No, audit tool - User-driven: Clinician - Risk score: Yes	Randomised feasibility study - Quantitative usage data - Qualitative interviews	**Quantitative** - Data related to tool use (symptoms entered and risk score generated) - Individual patient data from EMR 6 months after the index consultation, including data on secondary care procedures and diagnoses **Qualitative** GP interviews re facilitators and constraints influencing implementation of eCDS in routine practice	Use of eCDS by GPs was very low and only loosely consistent with use claimed during interviews Problems with use were identified by all GPs interviewed ‘lack of integration of the software with the clinical systems’ (*n* = 7) ‘slow to access and/or use’ (*n* = 6) ‘You had to open up something completely separate to the clinical system that you’re working in, and when you’ve got very very limited time that was a negative almost pushing you to not using it’ S Not enough time within consultations (*n* = 5) - ‘Patients never come with one symptom or issue, they come with a few different things, and we won’t automatically think, out of three problems, one of them is related to a gastric or oesophageal cancer, erm, I’m not necessarily going into the tool’. (GP5) - ‘No way on this planet any of the GPs under the pressure we were under(…)was going to use a separate program’	Perception **Impact on time**: Increase **Driving perception**: limited time in consultations, separateness from the clinical system, time needed for coding, and complexity of consultations
Scheitel et al. 2017 [120]	USA	To assess the impact of our clinical decision support tool on the efficiency and accuracy of clinician calculation of cardiovascular risk and its effect on the delivery of guideline-consistent treatment recommendations	**Condition of focus:** Cholesterol management **Setting**: Primary Care **Tool**: Cardiovascular risk scores and guideline-based treatment recommendations - Embedded/linked with EMR: Yes - Interruptive alert: No, audit tool - User-driven: Clinician - Risk score: Yes	Quantitative: - usage data - survey data	Time spent making calculation and recommendation Efficiency of clicks and key strokes making calculation and recommendation Accuracy of calculation and recommendation Survey Results	Without the tool, clinicians spent an average of 4 min and 21 s to calculate ASCVD score and a total of 5 min and 8 s to additionally determine care/treatment With the tool, the clinicians spent 39 s to calculate ASCVD score and a total of 1 min and 31 s determine a recommendation for patient care The **clinicians saved 3 min and 42 s in calculating ASCVD score and a total of 3 min and 38 s in determining care/treatment**. The time savings were statistically significant	Objective measure of time to use tool **Impact on time:** Decrease of 3 min 38 s
Seol et al. 2021 [121]	USA	To assess the effectiveness and efficiency of intervention via CDS on pertinent asthma outcomes in a real-world primary care setting	**Condition of focus:** Asthma **Setting**: Paediatric Primary Care **Tool**: CDS for asthma guidance and prediction to predict risk of adverse events (A-GPS) - Embedded/linked with EMR: Yes - Interruptive alert: No, audit tool - User-driven: Clinician - Risk score: Yes	Quantitative: notes review and survey data	**Primary**: adverse event (AE) within 1 year (ED visit/hospitalisation for asthma or unscheduled visit for asthma requiring oral corticosteroid) **Secondary**:- - Clinician burden for reviewing and collecting clinical data from EMR for making a clinical decision - Healthcare cost - Asthma control status - Timeliness of asthma follow up care after AE	A-GPS significantly reduced clinician burden for chart review for asthma management by 67%, with an estimated median time to review patient’s medical records of 3.5 min (IQR: 2–5) with A-GPS intervention vs. 11.3 min (IQR: 6.3–15) without A-GPS (*P* < 0.001). Average decrease within a person with A-GPS (vs. without A-GPS) was 7.3 min	Objective measure of time to use tool **Impact on time**: Decrease of 7.3 min
Shillinglaw et al. 2012 [97]	USA	To examine US physicians’ awareness, use, and attitudes regarding global CHD risk assessment in clinical practice, and how these vary by provider specialty	**Condition of focus:** Chronic Heart Disease **Setting**: Primary Care **Tool**: CHD risk assessment tools - Embedded/linked with EMR: Range - Interruptive alert: No - User-driven: Clinician - Risk score: Yes	Quantitative survey	Awareness of tools available to calculate CHD risk Method and use of CHD risk assessment Attitudes towards CHD risk assessment Frequency of using CHD risk assessment to guide recommendations of aspirin, lipid-lowering and blood pressure (BP) lowering therapies for primary prevention	Reasons for not using CHD risk assessment: - Among physicians who reported not using CHD risk assessment (*N* = 492), the reason with the highest mean importance rating was, “**It is too time consuming**" - Family physicians rated this reason higher than general internists and cardiologists	Perception **Impact on time**: Increase **Driving perception**: limited time in consultations
Siaki et al. 2021 [122]	USA	To determine the feasibility of a web-based clinical decision support tool (CDST) using a renin-aldosterone system (RAS) classification matrix and drug sequencing algorithm to assist providers with the diagnosis and management of uncontrolled hypertension	**Condition of focus:** Hypertension **Setting**: Primary Care **Tool**: CDS using a renin-aldosterone system classification matrix and drug sequencing algorithm to support diagnosis and management of uncontrolled HTN - Embedded/linked with EMR: No - Interruptive alert: No - User-driven: Clinician - Risk score: No	Quantitative data on hypertension clinical measures + survey of clinicians	**Primary**: 1) BP rates of control 2) clinician management time using an electronic logbook 3) Satisfaction with the CDS	The fastest clinician averaged 10 min per patient. The slowest took 20.56 min The overall average was 16.59 min (17.05% less time spent), saving 3.41 min per office visit avoided	Objective measure of time of whole consultations **Impact on time**: Decrease
Takamine et al. 2021 [123, 124]	USA	To understand providers’ views on the opportunities, barriers, and facilitators of incorporating risk prediction to guide their use of cardiovascular preventive medicines	**Condition of focus:** Cardiovascular disease **Setting**: Primary Care **Tool**: CVD risk assessment tools - Embedded/linked with EMR: Range - Interruptive alert: No - User-driven: Clinician - Risk score: Yes	Qualitative interviews	Attitudes toward adoption of an ASCVD risk prediction-based approach Key provider concerns - Quantified risk goals vs a “whole patient” approach - Validity of risk prediction - Compatability with workflow - Does adopting risk prediction add value? The role of performance measurement	**Compatability with workflow** - Given time pressures, concerns about workflow were common. 'You’re going to create a reminder in there, what else are you going to take off my plate?’ **Cognitive burden** - A few mentioned cognitive burdens associated with switching to this novel method: it’s easier to treat an A1C down to a certain value than, “Well, if this person’s A1C is 7.5, the cardiovascular risk is a certain number, and if we get it down to 6.8, the cardiovascular risk is a different number.”’	Perception **Impact on time**: Increase **Driving perception**: limited time in consultations, time needed to calculate or search for risk numbers, adding work without reducing anything else
Wan et al. 2012 [125]	Australia	To evaluate the uptake and use of the CDS tool as well as to describe the impact of the EDS tool on the primary care consultation for diabetes from the perspectives of general practitioners and practice nurses	**Condition of focus:** Diabetes **Setting**: General Practice **Tool**: CDS to support management of patients with type 2 diabetes - Embedded/linked with EMR: Yes - Interruptive alert: Yes - User-driven: Clinician - Risk score: Yes	Qualitative interviews	Use of the CDS tool Impact of the tool on the consultation process Impact of the tool on diabetes care Barriers to the use of the tool Suggestions on its improvement	**Impact on the consultation process** - GPs’ perceptions varied on the impact of the tool on consultation times. Many felt they tended to spend longer with patients when using the tool compared to usual consultations, but that this was because they were using the tool to provide better quality of care for their patients - One PN felt the consultation was lengthened, and another reported no change - Problems with tool functionality were perceived by some GPs as costing time. Some thought the tool itself slowed down their IT systems - Some felt the tool was distracting when the patient wasn’t attending for diabetes care	Perception **Impact on time**: mixed views **Driving perception**: limited time in consultations, tool is time consuming and distracting, better quality care takes longer
Wright et al. 2020 [126]	New Zealand	To assess the acceptability and feasibility of the Maternity Case-finding Help Assessment Tool (MatCHAT), a tool designed to provide e-screening and clinical decision support for depression, anxiety, cigarette smoking, use of alcohol or illicit substances, and family violence among pre- and post-partum women under the care of midwives	**Condition of focus:** Perinatal mental health **Setting**: Midwifery **Tool**: CDS for screening for antenatal and postnatal depression, anxiety, substance use and partner violence - Embedded/linked with EMR: No - Interruptive alert: No - User-driven: Clinician - Risk score: Unclear	Mixed methods: Quantitative usage data + qualitative interviews with midwives	**MatCHAT usage data** - numbers of screens completed - positive cases - participants who wanted help and the level of care recommended - survey ratings of acceptability, feasibility and utility **Interviews** - the MatCHAT prototype - midwives' knowledge - barriers to implementation	**Barriers to implementation** - midwives unanimous that MatCHAT was ‘one more thing’ in their hectic work schedule, and this influenced the low uptake - The midwives who did not use MatCHAT thought that it would increase the length of appointments: ‘I was in conflict because I know I needed to ask those questions and MatCHAT would have been useful for that, but in another way, it was going to take up a large chunk of time.’ - midwives also worried that screening might ‘open a can of worms’	Perception **Impact on time**: mixed views **Driving perception**: - increase: heavy workload and limited time, worry that tool would increase time, might 'open a can of woms' - decrease: if used efficiently can cut overall time spent screening
Wu et al. 2013 [127]	US	To examine physicians' experiences of using MeTree, a computerized Family Health History CDS tool which includes risk stratification	**Condition of focus:** Family health history **Setting**: Primary Care **Tool**: a CDS tool to collect data on family health history and risk stratification for various conditions	Mixed methods: Quantitative survey + qualitative interviews with physicians	**Survey and interviews:** Ease of integration of MeTree into clinical practice at the two intervention clinics	Physicians initially felt that they were already collecting high-quality family histories and that MeTree would negatively impact workflow and redirect prioirities However, post MeTree introduction, 86% of physicians believed that the tool improved the way they practised medicine, thereby making practice easier, and none reported that it adversely affected their workflow	Perception **Impact on workflow**: No negative impact **Driving perception**: the tool improved the way they practised, making practice easier
Dexheimer et al. 2013 [128]	US	To examine whether an automatic disease detection system increases clinicians’ use of paper-based guidelines and decreases time to a disposition decision	**Condition of focus:** Asthma **Setting**: Primary Care **Tool**: a computerized asthma detection system combined with a paper-based asthma care protocol in the pediatric ED to help standardize care and reduce time to disposition decision	Quantitative data on time to disposition decision	**Workload and efficiency outcomes assessed:** time for disposition decision in the ED as the primary outcome	No effect from the use of eCDS on the time taken by the ED physicians to make a disposition decision	**Objective measure of time** **Impact on time:** Neither increase or decrease
Moffat et al. 2014 [129]	UK	An evaluation my CRUK of cancer CDS tools	**Condition of focus:** Cancer **Setting:** General Practice **Tool:** eCDS using cancer risk algorithms with an interruptive risk score alert, risk calculator and audit function	Mixed methods: Quantitative usage data of tool and referrals + qualitative interviews with GPs	**Quantitative data:** use of the tools in practice, referrals **Qualitative:** impact on practice and the management of patients, and considerations and implications for further work in this area	Interviews with GPs highlight the varying impact of the tools on practice, ranging from no impact at all, to increasing knowledge, to influencing the management, including referral or investigation, of patients GPs were concerned about the level at which the prompt was set (i.e. at what level of risk a prompt appeared on their screen) and the potential for ‘prompt fatigue’ Some GPs expressed concerns that a 10-min consultation was a barrier to use of the symptom checker function within the tool	Perception **Impact on time**: mixed views **Driving perception**: Increase: concern regarding risk threshold of the alert and lack of time within a 10-min appointment to use the tool Decrease/no impact: Use of the tool did not influence the decision to investigate or refer in the majority of cases
Murphy et al. 2012 [130]	US	To measure the time spent by physicians managing asynchronous alerts	**Condition of focus:** generic **Setting:** Primary Care **Tool**: Asynchronous alerts during the day regarding a range of conditions	Quantitative: time spent processing alerts		On average, clinicians received over 56 alerts per day and spend 49 min responding to asynchronous alerts. These alerts burden clinicians in terms of physical fatigue and cognitive weariness. Providing clinicians with protected time to respond to alerts reduces alert-related workload and improves patient safety	Objective measure of time spent processing alerts **Impact on time**: No conclusion

### Workload-related findings

The scoping review had the broad aim of identifying evidence regarding impacts on workload and workflow; evidence most frequently reported these issues in terms of time and consultation durations. Findings from articles relating to perceived and objectively-measured impacts on either the time spent interacting with an eCDS tool or on whole consultation durations are summarised first (also in Table 2). Findings from articles that reported other workload-relevant results are summarised after.

**Table 2:** Summary of key findings from qualitative and quantitative evidence

Durations of consultations when using eCDS tools	Potential explanatory factors highlighted
Perceived duration (mainly qualitative studies) *n* = 72	
Perceived increase in duration (*n* = 36) [15, 16, 27, 28, 30–32, 34–37, 41, 44, 47, 50, 53, 61, 71, 77, 78, 83, 89, 92, 93, 96, 97, 101, 102, 104, 107, 110, 112, 115, 117, 124]	Existing time/workload pressures [16, 27, 28, 30, 31, 36, 41, 44, 47, 50, 71, 77, 78, 89, 93, 96, 97, 101, 104, 107, 110, 112, 115, 117, 124, 131]; eCDS tools will add burden [92, 104] No time for preventive care [32] Workflow disruption [30, 83, 93, 102, 104]; interruptive alerts/functions [117] Slow software [35, 37, 61]; being a separate system to EMR [16, 34, 71] Change to the trajectory of conversation with patient [15, 53, 104] Increased consultation duration might be ‘acceptable’ in some cases
Perceived decrease in duration (*n* = 6) [62, 67, 67, 131]	eCDS tools that were seen to improve efficiency [62, 69, 81] Tools designed to support patient management (rather than diagnosis) [69, 81, 98, 118] Tools that were embedded in the EMR [62, 81, 118] Reduced need for data entry [81, 98] Fitting with usual workflow [118]
Perception of no impact on duration (*n* = 4) [42, 63, 114, 127]	No obvious explanatory factors highlighted Low number of instances where a cardiovascular (CV) risk eCDS tool indicated high risk [63] Tools that guide the whole consultation and that were non-interruptive [42, 108, 127]
Objectively-measured duration (mainly quantitative studies) *n* = 26	
Increased duration (*n* = 3) [38, 54, 117]	An eCDS tool which took longer than the length of a typical consultation [54]
Decreased duration (*n* = 4) [116, 120–122]	eCDS tools that helped speed up certain tasks, e.g. calculating CV risk [116, 120] and clinical decision-making, asthma chart review [121] Tools designed to support management rather than diagnosis [116, 121, 122]
No impact on duration (*n* = 9) [40, 48, 57, 84, 95, 103, 109, 119]	Fitting in with usual workflow ^100^ Low usage of the study tool

Keywords identified from initial search:

•General practice / primary care

•Decision [making] [support]

•Computer / Online / Electronic

•Tool / System / Prompt

•Risk [assessment]

•Consultation / appointment

#### Perceived impacts on consultation duration

Seventy-two articles described impacts on consultation duration. These were gathered from qualitative interviews or focus groups with health professionals, often with the aim of identifying barriers and facilitators to implementing eCDS tools in practice. In spite of the wide range of contexts and functionalities of eCDS tools encompassed within this review, the majority of articles indicated that using an eCDS tool was thought to be associated with an increase in consultation duration (*n* = 36). Some showed a mix of views among health professionals (*n* = 20). Six articles reported an overall impression that an eCDS tool reduced or ‘saved’ time within the consultation. The remaining articles either indicated no perceived impact on consultation duration (*n* = 4) or made no explicit conclusion (*n* = 7).

### Perceived increase in consultation duration

Among the 36 articles that indicated a perceived increase in consultation duration, the most commonly highlighted concerns related to existing time pressures and lack of time during a consultation for clinicians to interact with eCDS tools and/or to carry out resultant recommended actions [16, 27, 28, 30, 36, 41, 47, 76, 93, 96, 97, 100, 102, 110, 111, 115, 123]. A prevalent view was that workload was ‘already heavy’ and that using eCDS tools would inevitably add burden [31, 44, 49, 60, 70, 78, 89, 102, 111, 115, 123]. In the case of one tool to support delivery of preventive care through review of patients’ lifestyle factors, the sense of lack of time for preventive care in general drove the view towards the tool increasing consultation duration [31]. Hirsch et al. (2012), however, highlighted that even though the majority of physicians in their study subjectively appraised consultation duration as being extended (85%), there were more of these physicians who felt that the time extension was ‘acceptable’ than those who judged it to be ‘unacceptable’ [52].

The usual flow of tasks to complete during a consultation (often referred to as ‘workflow’ [29, 33, 35, 39, 40, 42, 47, 58, 66, 67, 72, 74, 75, 84, 86, 91]) was commonly expected to be disrupted by eCDS tools, causing an increase in consultation duration [30, 34, 35, 47, 83, 93, 102, 104]. Specific time-consuming functions of tools, such as reading text, additional data-entry and using tools which were stand-alone from the EMR [16, 34, 70, 92, 102, 107, 117, 123], as well as perceptions of poor- or slow-functioning software [35, 37, 60, 104] were also highlighted. A potential for negative impact of eCDS tools on the trajectory of the conversation with patients was expressed by some health professionals. Some expressed concerns that introducing unexpected discussion, such as addressing the risk of cancer, would overtake the allotted consultation time and cause clinics to run late [15, 33, 53, 92].

Among these 36 articles, a wide range of eCDS tools with varying features and functionality were described (some overlapping). Thirteen involved tools which could interrupt the consultation, by presenting an on-screen alert containing risk or safety information, triggered by opening the EMR or by inputting diagnosis or prescription details [27, 28, 36, 53, 59, 78, 83, 89, 92, 93, 100, 104, 117, 131]. In addition, ten of these articles specifically highlight the issue of the tool directing the clinician’s attention towards a condition or matter that was not the reason for the encounter [27, 28, 33, 36, 53, 60, 63, 83, 100, 102, 104]. This was seen as necessitating additional time and/or workload, as a result of requiring prolonged discussion with the patient, serving as a distraction, and adding more tasks to already busy consultations. An eCDS tool flagging an issue that did not match the reason for the encounter could be unhelpful if seen as an ‘unwelcome intrusion’ [105], or if undermining a clinician’s professional expertise (particularly if there are doubts regarding the tool’s accuracy [51]) [105]. Such perceptions would be barriers to using or responding to such tools [51, 53, 60, 104]. Arranging a follow-up consultation in order to allow time for additional discussion and tasks was cited as an option for overcoming such barriers [27, 33].

Thirteen articles presented non-interruptive eCDS tools, accessed by a clinician at any time, used to obtain information, decision support or risk calculation, either for individual patients or as an audit tool used across the practice population [15, 30–35, 37, 41, 44, 49, 97, 123]. Eight articles described systems that were standalone from the EMR such as web-based eCDS tools [15, 16, 31, 32, 34, 37, 44, 49].

### Perceived decrease in consultation duration

The six articles that reported a perceived decrease in consultation duration suggested explanations which included reduced need for data entry [62, 81, 98], synchronisation with the usual workflow of decision-making [118] and saving time when discussing risk management of specific conditions during the consultation [67, 68]. In terms of the purpose, feature and functionality of the studied eCDS tools, the articles referred mainly to tools that were seen to improve efficiency, four of which featured a tool designed to support clinicians in the management of conditions, rather than on their diagnosis. All of the tools described were either embedded within the EMR system or linked/interacted with the EMR in some way. Two included an interruptive component among other functions [67, 68] and two were entirely user-accessed [62, 81].

### No perceived impact on consultation duration

No specific causal factors were suggested by the articles that reported an overall perception of no impact on consultation duration. One study of a cardiovascular risk assessment tool highlighted that consultation duration was perceived to be increased in cases where the GP did not expect the patient’s risk to be high, however the number of such instances was low [63]. A study involving both a survey and interviews with US physicians about a family history data collection tool showed that none reported an adverse impact on their workflow [127]. In terms of the studied eCDS tools’ purpose, features and functionality, the tools described included one with an interruptive component (cardiovascular risk score alert [63]) and two that were non-interruptive: a tool pre-populated by clinic staff that generated an email to the physician one week ahead of a patient’s visit to prioritise Chronic Kidney Disease care [42], and a computerised Family Health History CDS tool which included risk stratification [127].

#### Objectively-measured impacts on consultation duration

Twenty-six articles reported an objective measure of time. These included: (i) time spent using or interacting with an eCDS tool (ranging from three seconds [73] to between 0.5–13 min [35, 50, 54, 84, 90, 116, 120, 121]) and/or (ii) consultation duration [30, 38, 40, 45, 48, 57, 73, 79, 95, 103, 108, 109, 113, 114, 119, 122], including one which measured time from triage to final disposition decision [128].

### Increase in consultation duration

Overall, three articles suggested that consultation duration increased, although none measured consultation duration directly. Two of these articles reported that the time taken to use the eCDS tool was ‘too long’ for a typical ten-minute consultation (four minutes [50] and 13 min [54]), implying that consultation durations would increase as a consequence. One of these two articles highlighted the low rates of usage of the eCDS tool as an important consideration alongside the authors’ conclusion [49]. The third study also did not directly measure time, but instead reported ‘visit type’ as a proxy measure of consultation duration; clinicians more often used the eCDS tool in the longer, annual medical review visits (usually allotted 40 min in that study) than in the shorter, acute care visits [38].

No particular purpose, features, or functionality were shared by the eCDS tools described in these articles. In addition, none were highlighted as potential explanatory factors for the concluded increase in consultation duration.

### Decrease in consultation duration

Four articles suggested that consultation duration decreased, noting that the eCDS tools helped clinicians to undertake specific tasks more quickly. Two found that calculating cardiovascular risk scores and making clinical decisions, when assisted by an eCDS tool, was faster [116, 120], and another found a 7.3-min reduction in time within an asthma chart review consultation [121]. The fourth reported consultations to be 3.41 min shorter on average when using an eCDS tool to support diagnosis and management of hypertension [122]. All of the tools featured in these articles supported clinicians in the management of long-term conditions by design, or included an element of management support, as opposed to solely supporting initial risk assessment and/or diagnosis. All bar one described tools that were embedded with the EMR system, with only one of these having an interruptive component [120].

### No impact on consultation duration

Nine articles concluded that eCDS tools neither extended nor saved time in consultations. Having compared an intervention and control group or a set of baseline and intervention consultations, five articles reported no significant difference in consultation duration [40, 103, 108, 109, 128]. Lafata et al. (2016) found no association between use of a range of eCDS tools with the consultation duration. [57] The remaining articles reported that their measure of duration when using various eCDS tools (9.05 min [48] and 10 min [95]) was ‘similar’ in length to a standard consultation, concluding that the tools did not prolong consultations [119]. The remaining articles did not make any stated conclusion regarding duration or the conclusion was unclear [79, 84, 90, 114, 130].

A common explanation for lack of impact on consultation duration, or where perceptions of such impacts were mixed, was low rates of tool usage by clinicians in studies. Suggested reasons for non-use included perceived or actual difficulties in the tool’s functionality, slow-functioning software [30, 35, 37, 61], disruption to the usual workflow in a consultation [30, 83, 93] or requiring additional data entry to what would normally be inputted to the EMR, particularly where eCDS tools operated as a standalone system [34, 71].

In terms of purpose, features, and functionality of the tools described by these articles, while one article discussed only a stand-alone system from the EMR [95], the other articles reported either a tool embedded in the EMR system or described a range of both embedded and stand-alone systems. None of the described tools had an interruptive component. Most were guiding or supporting either prescribing tasks or decision making during consultations with a focus on patient management.

#### Conflict between perceived and objectively-measured impacts on consultation duration

Seven articles reported both perceived and objectively-measured impacts on consultation duration of using eCDS tools. Two found that both their perceived and objective measures suggested increased duration [50, 114]. However, five indicated a conflict between the perceived and objectively-measured impacts [30, 35, 45, 73, 108]. The common perception was that consultation duration was (or would be) increased, but there was actually no measurable difference in duration found. All of the tools described by these five articles were embedded with the EMR system, and did not include an interruptive alert feature or pertain to conditions or tasks likely to be irrelevant to the consultation.

Trafton et al. (2010) described physicians’ perceptions that eCDS for prescribing opioid therapy was ‘too time-consuming’, with insufficient time available during a 15-min consultation to use it [73]. However, the measured time spent using the tool ranged from 3 s to 10 min, and the study concluded that clinicians had ‘a reasonable amount of time’ to use the system. Curry & Reed (2011) reported that physicians felt the time taken for an eCDS system to interact with the EMR was ‘too slow’ despite the captured duration for this interaction being less than one second, although it is unclear whether this reflects physicians’ views of the overall interaction time rather than data processing time specifically [35]. Bauer et al. (2013) reported that although primary care clinic staff felt that a paediatric visit eCDS system slowed down clinics, an “informal” time study did not show any significant delays [30].

Porat et al. (2017) reported that 13 GPs (38%) felt their consultations took longer when using an eCDS system. They felt that inputting free text into the EMR instead was faster, and these same GPs did indeed have longer consultations when using the tool (an average of 15.45 min compared with their baseline 13.53 min average consultations). However, this was the case only for the GPs who expressed concern about time, and not for the GP sample as a whole where no significant difference in consultation duration was observed.

Further, a study by Gregory et al. (2017) found that the perception of physicians regarding the time available to manage eCDS alerts (termed ‘subjective workload’) was not correlated with actual hours spent managing alerts based on physicians’ self-report (‘objective workload’) [46]. When the authors examined whether these ‘subjective’ or ‘objective’ workload measures predicted physician burnout, only the ‘subjective’ measure was predictive. This suggests that the perception of eCDS alert burden in the context of existing high workload is more problematic than the measure of actual time spent managing alerts.

#### Methods utilised to measure consultation duration

A range of methods was utilised to measure objectively consultation duration or the time spent using an eCDS tool. In five articles, clinicians provided a self-report of time spent, using either a paper or electronic case report form [45, 95, 114, 121, 122]. A member of the research team manually timed the duration of study consultations or scenarios in four articles. [40, 54, 57, 73] Five articles reported time data captured electronically from log files within the eCDS tool itself, including clinician time spent using particular elements of the tool or completing certain activities [35, 50, 73, 84, 90]. Three articles described using specialist software, operating in the background, designed to record users’ interactions with the eCDS tool during consultations [116, 119, 120]. Specific software included Morae Recorder and Camtasia, both TechSmith Corporation products. Three studies used video- or audio-recordings to capture consultation durations in addition to other elements of the consultation they aimed to observe [48, 80, 113]. Two articles that referred to the same core UK study, described capturing duration data from the practice IT system (Vision), based on the opening and closing of the EMR [108, 109]. One USA study estimated consultation duration based on the reasons patients were attending – either for a ‘shorter’ visit, such as for acute care or follow-up, or for a ‘longer’ visit, such as for a general medical examination [38], and two articles provided insufficient details of the methods used [30, 117].

### Other workload-related findings

Twenty-seven articles included additional workload-related findings. Twenty-three of these reported the impact on ‘workflow’, regarding how eCDS tools altered the usual order in which patient-related tasks were carried out [33, 35, 39, 40, 47, 58, 66, 74, 75, 83, 84, 87, 91, 93, 94, 100, 102–104, 111, 113, 118, 119, 127]. Five referred to the impact of using eCDS tools on the trajectory of dialogue with patients, to the extent that follow-up appointments were arranged to avoid consultations running late [15, 39, 75, 94, 100]. One of these mentioned clinicians’ concerns about ‘taking time away’ from other waiting patients, expressed as a barrier to the implementation of eCDS systems [26]. Many of the tools in these articles were clearly described as having an interruptive alert component [33, 58, 83, 84, 86, 91, 93, 100, 104, 111, 118, 119].

Some articles (*n* = 10) mentioned ‘alert fatigue’ indicating that eCDS tools designed to support health professionals can increase the number of on-screen alerts, leading to a high chance of them being missed or ignored [15, 36, 40, 42, 51, 74, 100, 105, 111, 117]. None of these articles reported a decrease in consultation duration.

Cognitive workload was referred to in three articles. Qualitative interview data suggested that clinicians felt an eCDS tool for prescribing tuberculosis preventive therapy decreased their cognitive workload during consultations. [98] This was perceived as advantageous as it reduced the amount of time spent documenting medications and their contraindications. However, in two articles, eCDS tools were noted to increase cognitive workload. A systematic review that examined factors influencing the appropriateness of interruptive alerts found such alerts increased cognitive weariness, and that an ‘overload’ of alerts increased mental workload [117]. A study of an eCDS tool for assessing cardiovascular risk also highlighted clinicians’ concerns about the cognitive burden of changing to a new way of calculating risk compared with the conventional method they had used until that point [124].

One study reported workload expressed as the number of follow-up consultations needed. This study examined eCDS tools for patients with upper respiratory tract infections, and found no significant difference in the proportion of follow-ups needed between the intervention and control arms [82].

## Discussion

This scoping review identified 95 articles that examined the use of eCDS tools by health professionals in primary care and reported findings that included impacts on workload and workflow. While the scoping review had the broad aim of identifying evidence regarding these issues, they were most frequently reported in terms of time and consultation durations.. A large proportion of the research was qualitative and exploratory in nature. The majority of articles reported health professionals’ subjective perceptions of time spent using eCDS tools and/or the impact on consultation duration and there was a smaller evidence base which objectively-measured impact of using eCDS tools on workload, specifically in relation to consultation duration and the flow of consulting sessions.

The reviewed literature reflected that although a small number of articles suggested that using certain types of eCDS tool decreased consultation duration, a strong perception exists among health professionals that consultation duration was increased when eCDS tools were used. It is worth noting that eCDS tools designed to support management of health conditions and tools supporting diagnosis and associated risk assessment may have different impacts on consultation workload and duration; the small number of reviewed articles that indicated a time saving mostly featured tools designed to support patient management. It is also notable that many of the articles describing tools that introduced a condition or issue that was outside of the patient’s or clinician’s agenda for the consultation, frequently reported clinicians’ perceptions that workload and/or consultation duration increased.

The perception that consultation duration was increased is not necessarily backed by studies that objectively measured actual durations of consultations. Although many of the quantitative articles reported the time taken to use various eCDS tools within consultations, fewer studies captured the duration of entire consultations and/or made a comparison between an intervention and non-intervention group. Interestingly, those that did showed no significant difference in consultation duration when using eCDS tools compared with not using them [40, 103, 108, 109, 128]. Various methods were used to capture consultation durations, with no one method that seemed most practical or accurate. For instance, while the manual (stopwatch) timing of consultations by a researcher [54, 73] might arguably capture consultation durations more accurately than clinicians’ self-report, this method could be seen as intrusive to the consultation. Capturing time stamp data in an automated way, for example from EMR systems [108, 109], might address this issue and provide a practical solution, but errors may be introduced by this method if patient records are left open after the end of a consultation, or some part of the consultation takes place when records are closed.

The reviewed literature highlighted that low usage rates of eCDS tools by clinicians in studies (for varying reasons) may be responsible for a lack of observable impact on workload or consultation duration. Conversely, a tool that fits easily within the usual workflow of a consultation might explain the lack of increased duration. The experience of ‘alert fatigue’ was frequently mentioned; a large number of different on-screen alerts during consultations can desensitise clinicians to alerts, and an alert generated by a new tool may be missed or ignored [27, 28, 50]. Ignoring an alert or not utilising an eCDS tool might indicate clinician’s preference to rely on their own clinical judgment, or doubts as to an alert’s accuracy or relevance, which is particularly highlighted within the alert fatigue literature [36, 107, 132–134]. It might equally be the case that a clinician did indeed utilise or respond to the eCDS tool, but arranged a follow-up appointment to allow for more time to discuss the clinical issues raised [26, 28, 33], thereby not impacting the duration of the current consultation. Whether use of eCDS tools had an impact on the duration of the healthcare ‘episode’ as a whole (i.e. the index consultation plus the number and duration of any subsequent follow-up consultations) was unclear from the reviewed articles.

Reviewing articles that included both a subjective measure of health professionals’ perceptions and an objective measure of consultation duration provided an opportunity to observe if the perceptions were borne out in reality. These articles most commonly reported that health professionals felt consultations were (or would be) prolonged by using eCDS tools, but objective measures did not consistently back this up [30, 35, 73]. However, the evidence base for actual consultation durations associated with using eCDS tools remains a lot smaller than that of the perceived impacts on consultation durations. One should note that the perception or expectation of health professionals in relation to consultation workload and duration is very important. Firstly, perceptions and expectations may well determine how often eCDS tools are used. Secondly, ‘subjective’ workload (clinicians’ reported amount of time available to manage alerts), rather than ‘objective’ workload (the number of hours actually spent managing alerts), has been found to be predictive of physician burnout [45]. It is worth also noting, however, that a perception or an objective measure of increased workload or duration may not always be viewed negatively; for example, it may not matter how much consultation duration is increased (if it is) if diagnosis and/or management is improved [52].

### Strengths and limitations

This study benefits from undertaking a comprehensive literature review addressing a key area of primary care service provision, namely the interface between technologically enhanced service provision in the form of eCDS, and clinical workload and workflow. We successfully identified and summarised a large number of articles published from a variety of international settings.

The review may have been affected by the inclusion of names of specific eCDS tools within the search terms. This reflects research team members’ awareness of existing systems in UK primary care; tools not known to the authors may have been missed from the review. We identified a number of studies through systematic reviews that were not found through our initial searches, this suggests that our initial searches may have missed some relevant work. Inclusion of articles published in the last ten years, since 2009, may also have omitted potentially-relevant research on eCDS since its inception in the 1960’s, however we aimed to identify evidence from research articles based in modern-day primary care settings In addition, although the vast majority of international scientific literature is currently published in English, our exclusion of foreign language articles may have prevented fuller coverage of non-UK primary care contexts with different standards of consultation lengths, workload or workforce challenges, and policy expectations. The review also included a large number of qualitative articles, but time and resource issues prevented a full qualitative synthesis of these articles.

The two independent reviewers who undertook screening were not always the same two reviewers due to resource constraints, however EF undertook all stages of the review and had regular discussions with the small group of four ‘second’ reviewers. Only EF undertook data extraction and so details from included articles may have been affected by selection bias.

## Conclusion

This scoping review identified over 90 articles that explored the use of eCDS tools in primary care by health professionals in relation to aspects of workload, including consultation duration. Whilst the qualitative literature showed a strong perception among health professionals that eCDS tools increased workload and consultation duration, a smaller number of studies captured quantitative measures, which neither disputed nor supported this view.

eCDS tools designed to support GPs will continue to be introduced within primary care with the aim of assisting clinicians to diagnose and manage patients effectively. Despite the absence of strong objective evidence that using eCDS tools necessarily leads to increased (or decreased) consultation durations, the perceptions of additional time being taken within consultations, additional workload being generated, and workflow being disrupted, are barriers to implementation and routine use of eCDS tools, irrespective of their potential benefit in the diagnosis or management of patients.

Further quantitative evidence measuring actual consultation duration and GP workload is needed to confirm whether the reported concerns are justifiable, particularly in the time-constrained setting of primary care. Future efforts to implement potentially valuable eCDS tools need to take account of the context of increasing GP workload, workforce shortages and associated pressures, and the ongoing challenges generated in the wake of COVID-19.

## Data Availability

All data generated or analysed during this study are included in this published article.

## Abbreviations

CDS: Clinical decision support
eCDS: Electronic clinical decision support
EMR: Electronic medical record
GP: General practitioner
